# Stress biomarkers and child development in young children in Bangladesh

**DOI:** 10.1016/j.psyneuen.2024.107023

**Published:** 2024-06

**Authors:** Zachary Butzin-Dozier, Andrew N. Mertens, Sophia T. Tan, Douglas A. Granger, Helen O. Pitchik, Dora Il'yasova, Fahmida Tofail, Md. Ziaur Rahman, Ivan Spasojevic, Idan Shalev, Shahjahan Ali, Mohammed Rabiul Karim, Sunny Shahriar, Syeda Luthfa Famida, Gabrielle Shuman, Abul K. Shoab, Salma Akther, Md. Saheen Hossen, Palash Mutsuddi, Mahbubur Rahman, Leanne Unicomb, Kishor K. Das, Liying Yan, Ann Meyer, Christine P. Stewart, Alan E. Hubbard, Ruchira Tabassum Naved, Kausar Parvin, Md. Mahfuz Al Mamun, Stephen P. Luby, John M. Colford, Lia C.H. Fernald, Audrie Lin

**Affiliations:** a School of Public Health, University of California, Berkeley, CA, USA; b Division of Infectious Diseases and Geographic Medicine, Stanford University, Stanford, CA, USA; c Institute for Interdisciplinary Salivary Bioscience Research, University of California, Irvine, CA, USA; d Department of Pediatrics, Johns Hopkins University School of Medicine, Baltimore, MD, USA; e Duke University School of Medicine, Durham, NC, USA; f International Centre for Diarrhoeal Disease Research, Dhaka, Bangladesh; g Department of Biobehavioral Health, Pennsylvania State University, University Park, PA, USA; h EpigenDx, Inc., Hopkinton, MA, USA; i Department of Nutrition, University of California, Davis, CA, USA; j Department of Microbiology and Environmental Toxicology, University of California, Santa Cruz, CA, USA

**Keywords:** Stress, Child development, Cortisol, Alpha-amylase

## Abstract

**Background:**

Hundreds of millions of children in low- and middle-income countries are exposed to chronic stressors, such as poverty, poor sanitation and hygiene, and sub-optimal nutrition. These stressors can have physiological consequences for children and may ultimately have detrimental effects on child development. This study explores associations between biological measures of chronic stress in early life and developmental outcomes in a large cohort of young children living in rural Bangladesh.

**Methods:**

We assessed physiologic measures of stress in the first two years of life using measures of the hypothalamic-pituitary-adrenal (HPA) axis (salivary cortisol and glucocorticoid receptor gene methylation), the sympathetic-adrenal-medullary (SAM) system (salivary alpha-amylase, heart rate, and blood pressure), and oxidative status (F2-isoprostanes). We assessed child development in the first two years of life with the MacArthur-Bates Communicative Development Inventories (CDI), the WHO gross motor milestones, and the Extended Ages and Stages Questionnaire (EASQ). We compared development outcomes of children at the 75th and 25th percentiles of stress biomarker distributions while adjusting for potential confounders using generalized additive models, which are statistical models where the outcome is predicted by a potentially non-linear function of predictor variables.

**Results:**

We analyzed data from 684 children (49% female) at both 14 and 28 months of age; we included an additional 765 children at 28 months of age. We detected a significant relationship between HPA axis activity and child development, where increased HPA axis activity was associated with poor development outcomes. Specifically, we found that cortisol reactivity (coefficient −0.15, 95% CI (−0.29, −0.01)) and post-stressor levels (coefficient −0.12, 95% CI (−0.24, −0.01)) were associated with CDI comprehension score, post-stressor cortisol was associated with combined EASQ score (coefficient −0.22, 95% CI (−0.41, −0.04), and overall glucocorticoid receptor methylation was associated with CDI expression score (coefficient −0.09, 95% CI (−0.17, −0.01)). We did not detect a significant relationship between SAM activity or oxidative status and child development.

**Conclusions:**

Our observations reveal associations between the physiological evidence of stress in the HPA axis with developmental status in early childhood. These findings add to the existing evidence exploring the developmental consequences of early life stress.

## Highlights

• We measured stress biomarkers and development in young children in Bangladesh.
• We evaluated biomarkers from multiple stress systems.
• We used generalized additive models to flexibly assess correlations.
• Hypothalamic-pituitary-adrenal axis markers were associated with child development.

## 1 Introduction

There are more than 250 million children in low and middle-income countries who are at risk of failing to reach their developmental potential (Black et al., 2017). Chronic stress experienced in early childhood can increase risk for poor developmental outcomes later in life (Franke, 2014, Grantham-McGregor et al., 2007, McEwen, 2011). Chronic early-life stress has the potential to negatively affect multiple biological systems and interfere with learning and memory, dysregulate metabolism and sleep, and negatively affect mental health (Lu et al., 2016, National Scientific Council on the Developing Child, 2014). A stressor is any stimulus that evokes a biological response, which includes psychosocial stressors as well as physical stressors (Chu et al., 2023, Del Giudice et al., 2018, Yaribeygi et al., 2017). Many children are exposed to stressors, such as iodine deficiency, inadequate cognitive stimulation, and infectious disease (e.g., malaria, diarrhea), that are associated with developmental delay (Rana et al., 2022, Walker et al., 2011, Walker et al., 2007). Stress biomarkers may reflect a mechanism by which stressors contribute to subsequent developmental status. An improved understanding of how specific biomarkers are related to development may have implications on pediatrics as well as policy (Shonkoff et al., 2022). Evaluating these associations in early childhood is particularly important, as this is an effective time to intervene on developmental outcomes (Engle et al., 2011).

Children in low-income, rural communities face many biological and psychosocial stressors (Walker et al., 2007). Social and economic factors can lead to developmental differences in children, and developmental neuroscience demonstrates how early experiences can influence development (Engle et al., 2011). Poverty and other sociocultural factors can alter neurological functioning, brain structure, and child behavior, which can in turn affect developmental status (Walker et al., 2007). Although cumulative exposure to stressors leads to an increased risk of poor developmental outcomes, the impact of these exposures depends on their timing, co-occurrence, and an individual’s reactivity (physiologic response) to these stressors (Walker et al., 2011). These cumulative stressors are often measured through stress hormones (Engle et al., 2007).

Stress can be an adaptive or maladaptive response to challenging stimuli across multiple biological systems. Primary stress biochemical pathways include the hypothalamic-pituitary-adrenal (HPA) and sympathetic adreno-medullary (SAM) axes (Condon, 2018, Johnson et al., 2013). Both the HPA and SAM axes play a role in the stress response to infectious disease and malnutrition, which are commonplace in rural, low-income settings such as rural Bangladesh (Bourke et al., 2016, Godoy et al., 2018, Lu et al., 2016, Walker et al., 2011). The first major component of the stress responses is the HPA axis which is controlled by a negative feedback loop in which pro-inflammatory cytokines stimulate HPA activation, triggering the release of anti-inflammatory cortisol, a glucocorticoid, which in turn dampens HPA axis activity (Johnson et al., 2013). The cortisol response enables individuals to respond to challenging circumstances, and cortisol is involved in mobilizing biological resources for metabolic, sensory, and learning processes (Booth et al., 2000). The *NR3C1* gene encodes glucocorticoid receptors, and early life stress is associated with increased *NR3C1* methylation, leading to decreased expression of this gene (van der Knaap et al., 2014). Prolonged activation of the HPA axis and excess cortisol can lead to oxidative stress, which is an excess of reactive oxygen species relative to antioxidants (Aschbacher et al., 2013). Urinary F2-isoprostanes are the biomarkers most frequently used to measure oxidative status (Il’yasova et al., 2010, Montuschi et al., 2004, Pizzino et al., 2017). While oxidative stress can be harmful, reactive oxygen species play a critical role in the human body and immune function (Pizzino et al., 2017).

The second major component of the psychobiology of the stress response is activation of the SAM axis, which increases blood pressure and heart rate through the release of epinephrine and norepinephrine (Brietzke et al., 2012, McEwen, 2000). Whereas the HPA response to stress is linked with negative affect, distress, withdrawal, and being overwhelmed, the SAM response to stress is associated with increased engagement, cognitive effort, attentional focus, work, and arousal (Brietzke et al., 2012, McEwen, 2000). This axis also triggers the secretion of salivary alpha-amylase, a carbohydrate digestion enzyme that has been recently used as a salivary stress biomarker (Condon, 2018).

Researchers have evaluated individual differences in the psychobiology of the stress response and the consequences of these differences on early child development (Coe et al., 2003, Gunnar and Fisher, 2006, Kagan et al., 1987, Loman et al., 2010). Investigators have extended these research questions to assess the impact of various potential sources of stress including rural poverty (Fernald and Gunnar, 2009), maternal experience of intimate partner violence (Huth-Bocks et al., 2002, Levendosky et al., 2013), extreme neglect (Gunnar and Fisher, 2006, Reilly and Gunnar, 2019), parental divorce (Afifi et al., 2009), poor nutrition (Fernald et al., 2003, Fernald and Grantham-McGregor, 1998), and maternal substance use (Labella et al., 2021) on developmental outcomes in early childhood, although further research is needed to understand the mechanistic pathways through which these stressors can lead to developmental consequences. Correlational studies have indicated that poverty is associated with increased child cortisol, and a 2009 quasi-experimental study found that children from families who participated in a cash transfer program had lower cortisol compared to children from families who did not participate (Fernald and Gunnar, 2009, Schmidt et al., 2021). A 2003 study in Nepal as well as a 1998 study in Jamaica found that stress reactivity (salivary cortisol and heart rate) was associated with growth impairment (Fernald et al., 2003, Fernald and Grantham-McGregor, 1998).

Throughout decades of research on stress and development, several themes have emerged. First, the biobehavioral manifestations of chronic stress are heterogenous based on individual, familial, and community-level factors (Godoy et al., 2018). Second, differences in biological responses to stress (i.e. reactivity) are largely responsible for translating experiences into differential outcomes (Amstadter et al., 2016, Jiang et al., 2019, Schmidt et al., 2021, Wolf et al., 2018). Third, the social context of the family and quality of family care, including stimulating and nurturing behaviors, moderate the effects of exposures on development (Asok et al., 2013, Bernard et al., 2015, Booth et al., 2000, Fisher et al., 2007). Further evaluation of these associations in the context of low- and middle-income countries, where there is a large burden of both early-life chronic stress and poor developmental outcomes, as well as frequent exposure to inflammation and infection, may provide important insights in a high-risk population (Black et al., 2017).

Previous studies have evaluated child development in low-income, rural settings through a variety of methods, including the MacArthur-Bates Communicative Development Inventories (CDI), WHO motor milestones, and the Extended Ages and Stages Questionnaire (EASQ) (Fernald et al., 2012; Hamadani et al., 2010; Ndayizigiye et al., 2022; Tofail et al., 2018; “WHO Motor Development Study: windows of achievement for six gross motor development milestones.,” 2006). CDI, a parental-report measure of language development, has been validated for use in rural Bangladesh and has been adapted for this population (Hamadani et al., 2010; Tofail et al., 2018). The WHO gross motor development score enables the evaluation of child motor skills and has been validated for children ages 4–24 months across multiple countries globally (“WHO Motor Development Study: windows of achievement for six gross motor development milestones.,”; 2006). The EASQ involves a combination of parental-report and direct observation (Fernald et al., 2012, Ndayizigiye et al., 2022). Psychologists from the International Centre for Diarrhoeal Disease Research, Bangladesh (icddr,b) adapted this tool for use in Bangladesh by adding direct assessment to 25% of the evaluation items (Tofail et al., 2018). The EASQ has been used to accurately capture variation in child development across different levels of socioeconomic status and at-home child stimulation in low-income countries (Fernald et al., 2012, Ndayizigiye et al., 2022).

This study aims to evaluate the associations between markers of HPA axis activity, SAM axis activity, oxidative stress, and child development outcomes in a cohort of young children (assessed at median ages 14 and 28 months) in rural Bangladesh. We hypothesized that decreased oxidative status and decreased salivary alpha-amylase would be associated with higher child development scores, while higher salivary cortisol, higher glucocorticoid receptor methylation, and higher heart rate and blood pressure would be associated with higher child development scores (Supplemental Table 1).

**Table 1:** Descriptive statistics of sample population.

			n (%) or median (IQR)
Child		Female	761 (49%)
	Urinary F2-isoprostanes (ng/mg creatinine; Year 1)	iPF(2α)-3	-0.42 (-0.72, -0.09)
		2,3-dinor-iPF(2α)-III	1.76 (1.55, 1.97)
		iPF(2α)-VI	2.57 (2.31, 2.87)
		8,12-iso-iPF(2α)-VI	2.58 (2.17, 2.91)
	Salivary cortisol reactivity (μg/dl; Year 2)	Cortisol reactivity	0 (0, 0.01)
		Cortisol residualized gain score	-0.09 (-0.21, 0.14)
	Salivary alpha-amylase reactivity (U/ml; Year 2)	Salivary alpha-amylase reactivity	1.41 (-0.08, 4.41)
		Salivary alpha-amylase residualized gain score	-25.83 (-51.4, 28.68)
	Sympathetic-adreno-medullary biomarkers (Year 2)	Mean arterial pressure (mmHg)	64.44 (60.78, 68.78)
		Resting heart rate (bpm)	109 (99.33, 118.67)
	Glucocorticoid receptor percent methylation	*NR3C1* exon 1F promoter	-5.66 (-6.06, −5.32)
		NGFI-A transcription factor binding site	-4.48 (-4.65, −4.14)
	Child development (Year 1)	WHO gross motor milestone sum score	2 (1, 4)
		CDI expressive language z-score	0.02 (-0.54, 0.74)
		CDI language understanding z-score	0.09 (-0.56, 0.78)
	Child development (Year 2)	EASQ communication z-score	0.37 (-0.39, 0.75)
		EASQ motor development z-score	-0.15 (-0.59, 0.87)
		EASQ personal-social development z-score	0.14 (-0.49, 1)
		EASQ combined z-score	0.3 (-0.37, 0.86)
		CDI expressive language z-score	0.27 (-0.57, 0.8)
		CDI language understanding z-score	0.12 (-0.48, 0.74)
	Anthropometry (14 months, Year 1)	Length-for-age z-score	-1.42 (-2.07, −0.76)
		Weight-for-age z-score	-1.31 (-2.01, −0.64)
		Weight-for-length z-score	-0.9 (-1.56, −0.23)
		Head circumference-for-age z-score	-1.79 (-2.35, −1.13)
	Anthropometry (28 months, Year 2)	Length-for-age z-score	-1.56 (-2.28, −0.95)
		Weight-for-age z-score	-1.58 (-2.2, −0.94)
		Weight-for-length z-score	-1.03 (-1.62, −0.38)
		Head circumference-for-age z-score	-1.81 (-2.38, −1.21)
	Diarrhea (14 months, Year 1)	Caregiver-reported 7-day recall	195 (13%)
	Diarrhea (28 months, Year 2)	Caregiver-reported 7-day recall	110 (7%)
Mother		Age (years)	24 (20, 27)
	Anthropometry at enrollment	Height (cm)	150.2 (146.8, 154.05)
	Education	Schooling completed (years)	7 (4, 9)
	Depression at Year 1	CESD-R score	10 (6, 16)
	Depression at Year 2	CESD-R score	10 (5, 17)
	Perceived stress at Year 2	Perceived Stress Scale score	14 (10, 17.25)
	Intimate partner violence	Any lifetime exposure	810 (57%)

CDI: MacArthur-Bates Communicative Development Inventories; EASQ: Extended Ages and Stages Questionnaire; CESD-R: Center for Epidemiologic Studies Depression Scale Revised

## 2 Methods and materials

These analyses utilize data from the WASH Benefits study, described in detail previously (Luby et al., 2018, Tofail et al., 2018). The trial enrolled pregnant mothers in Bangladesh in their first or second trimester of pregnancy in rural subdistricts in Gazipur, Mymensingh, Tangail, and Kishoreganj and followed the cohort of children from birth until 2.5 years of age. Here, we describe observational analyses of the associations between child stress biomarkers and concurrent and subsequent child development in a subsample of children from the trial. Sample household enrollment characteristics remained similar to the main trial (Lin et al., 2020). This subsample included participants from four treatment arms: (1) control (no intervention, *n* = 395), (2) combined intervention on water, sanitation, and hygiene (WSH, *n* = 377), (3) nutrition (*n* = 347), and (4) nutrition plus WSH (*n* = 436) (Luby et al., 2018). The water intervention included provision of chlorine tablets for drinking water treatment and a water storage vessel. The sanitation intervention involved upgrading pit latrines for all households in study compounds, providing child potties, and providing scoops for waste disposal in or near the household. The hygiene intervention involved handwashing promotion which included the establishment of a handwashing station with soapy water and detergent soap. The nutrition intervention provided a lipid nutrient supplement and conseling training on child nutrition. This sample included 684 children aged 14 months (median age, Year 1) and 1449 children (49% female) aged 28 months (median age, Year 2).

### 2.1 Correlates of stress biomarkers

Child stress biomarkers included markers of the HPA axis, the SAM axis, and oxidative status. HPA axis biomarkers were salivary assessments of cortisol and glucocorticoid receptor (*NR3C1*) methylation. SAM axis measures were salivary alpha-amylase, resting heart rate, and mean arterial pressure. We measured oxidative status using four urinary F2-isoprostanes. We measured oxidative status markers at Year 1 (median age 14 months), while we assessed HPA and SAM axis biomarkers at Year 2 (median age 28 months; Table 1). We assessed resting heart rate and mean arterial pressure in four study arms, but we only measured other stress biomarker exposures in two arms, which led to a greater sample size at Year 2 compared to Year 1.

#### 2.1.1 Urinary F2-isoprostanes

We analyzed four urinary F2-isoprostane isomers separately (iPF(2α)III, 2,3-dinor-iPF(2α)III, iPF(2α)-IV, and 8,12-iso-iPF(2α)-VI), and we also used the first component of a principal components analysis of the four measures of urinary F2-isoprostanes (as these measures were correlated; P-value <0.2) to assess overall oxidative status (Jolliffe and Cadima, 2016). Investigators collected urine samples at Year 1 in Briggs Pediatric Sterile U-Bags and preserved samples with 0.1% thimerosal, and collected pooled aliquots over a period of five hours (protocol published elsewhere) (Lin et al., 2020). Duke University research team members evaluated four isomers of F2-isoprostanes [iPF(2α)-III; 2,3-dinor-iPF(2α)-III; iPF(2α)-VI; 8,12-iso-iPF(2α)-VI] in urine samples using liquid chromatography-tandem mass spectrometry (LC-MS/MS) and adjusted F2-isoprostanes concentrations for urine diluteness (Il’yasova et al., 2010).

#### 2.1.2 Salivary cortisol and alpha-amylase

We assessed stress reactivity at Year 2 as the change in salivary cortisol and salivary alpha-amylase following venipuncture. Acute physical stressors (blood draw, physical exam, etc.) are often used to elicit a stress response in young children (Jansen et al., 2010, Nater and Rohleder, 2009). In this setting, venipuncture serves as both a physical and psychological stressor, as it involves physical discomfort as well as the psychological stress of physical separation of the child and caregiver. We collected saliva five to eight minutes before the stressor onset, five minutes post-stressor, and 20 minutes post-stressor using SalivaBio Children’s Swabs (Salimetrics). We measured cortisol pre- and 20 minutes post-stressor, and measured alpha-amylase pre- and five minutes post-stressor (Granger et al., 2007a, Granger et al., 2007b). The icddr,b Mymensingh satellite laboratory evaluated sAA and cortisol (Salimetrics, Carlsbad, CA) with commercial ELISA kits. The caregiver was instructed not to allow their child to ingest caffeine or medicine for one hour prior to these study procedures, and we rinsed the child’s mouth with drinking water 15–20 minutes prior to the stressor. We calculated cortisol and alpha-amylase reactivity as the post-stressor value minus the pre-stressor value, divided by the time elapsed between samples. We recorded time of salivary biomarker assessment to account for circadian patterns of hormone production.

#### 2.1.3 Glucocorticoid receptor methylation

We collected saliva samples for DNA methylation analysis in Oragene kits (OGR-575). EpigenDx (Hopkinton, MA) extracted DNA from these samples, conducted bisulfite treatment and pyrosequencing, performed PCR, and determined percent methylation (Lin et al., 2021c) We assessed percent methylation across the entire glucocorticoid receptor (*NR3C1*) exon 1 F promoter (39 assayed CpG sites) as well as the nerve-growth factor inducing protein A (NGFI-A) transcription factor binding site, which is a specific site within the *NR3C1* exon that is associated with hippocampal glucocorticoid receptor expression (Edelman et al., 2012, Zhang et al., 2013).

#### 2.1.4 Resting heart rate and blood pressure

We measured resting heart rate and blood pressure in triplicate to ensure reliability at Year 2, where we included the median of the three measurements, and we assessed the mean arterial pressure as two times the diastolic blood pressure, plus the systolic blood pressure, divided by three (Curtis et al., 2015, Fagan et al., 1988, Rehman and Nelson, 2022). We measured resting heart rate using a finger pulse oximeter (Nonin 9590 Onyx Vantage) and blood pressure (systolic and diastolic) using a blood pressure monitor (Omron HBP-1300).

#### 2.1.5 Biomarker data transformation

We log-transformed F2-isoprostane, cortisol, salivary alpha-amylase, and glucocorticoid receptor methylation distributions to account for skewness.

### 2.2 Assessments of child development

Primary outcomes included child development outcomes measured via the MacArthur-Bates Communicative Development Inventories (CDI) at Years 1 and 2, the WHO gross motor milestones module at Year 1, and the Extended Ages and Stages Questionnaire (EASQ) at Year 2.

The CDI includes assessment of language expression and comprehension. WHO motor milestones include six indicators of motor development– sitting without support, hands-and-knees crawling, standing with assistance, walking with assistance, standing alone, and walking alone. Motor milestone attainment was analyzed as a sum score of the 2nd, 4th, 5th, and 6th milestones (1st and 3rd milestones excluded due to a high proportion of children skipping over attainment of these indicators) to assess cumulative attainment as well as through a time-to-event analysis to assess the rate of attaining each milestone, which is consistent with previous analyses of this measure of development (Tofail et al., 2018; “WHO Motor Development Study: windows of achievement for six gross motor development milestones.,”; 2006). The EASQ has five domains, but only three were used in this study due to logistical field-work constraints: child communication, gross motor development, and personal-social development. We also generated a combined EASQ score (Fernald et al., 2012, Singh et al., 2017, Tofail et al., 2018). We age-standardized both CDI and EASQ scores using the control group as the standard population in 2-month age bins using standard techniques (Tofail et al., 2018). Child age was determined from the caregiver reported birth date of the child, which was verified using vaccination cards when available.

### 2.3 Analysis

We used R (version 4.1.1) to conduct observational analyses nested within a randomized controlled trial in accordance with a pre-registered analysis plan (https://osf.io/hzb6m/) (Lin et al., 2021a). This analysis controlled for child age, child sex, and covariates that were significantly associated with the outcome of interest in each unique analysis. We selected covariates of interest that may plausibly confound each exposure-outcome relationship (i.e., may be an independent cause of both exposure and outcome). We prescreened each covariate of interest using a likelihood ratio test to assess potential relationships between each covariate and each outcome, where we included each covariate that yielded a p-value less than 0.2 and excluded covariates with little (<5%) variation in the study sample. We evaluated the association between each exposure of interest (e.g., stress reactivity at Year 2) and each outcome of interest (e.g., CDI comprehension score at Year 2) independently (Supplemental Table 1), as each association potentially required its own, unique set of adjustment covariates to reduce confounding.

We used natural smoothing splines to accommodate potential nonlinearity and summarized mean developmental outcomes across stress biomarker distributions after controlling for potential confounders and covariates of interest in accordance with our pre-registered statistical analysis plan (Wood et al., 2016, Lin et al., 2021b, Hastie and Tibshirani, 1995; FAN Lin et al., 2019). All adjusted analyses included child age and sex, and we screened the following covariates for potential inclusion: birth order, maternal age and education, food insecurity, household crowding, access to drinking water, household assets, prior growth, treatment arm, month of assessment, assessment time, and maternal depression, stress, and lifetime exposure to any type of intimate partner violence. Additional information regarding covariate screening and inclusion can be found in Appendix 1. We then plotted these general additive model curves along with simultaneous confidence intervals (Nychka, 1988). These generalized additive models allowed us to flexibly assess non-monotonicity of relationships (Wood et al., 2016, Lin et al., 2021b, Hastie and Tibshirani, 1995). The primary contrast was the difference in the mean outcome at the 75th and 25th percentile of each exposure level after adjusting for relevant covariates, which we describe as “adjusted difference” hereafter (Lin et al., 2021b). Once we assessed monotonicity of exposure-outcome relationships, these adjusted differences (comparing binary exposure status) allowed us to generate interpretable estimates in a pre-specified manner (Wood et al., 2016, Lin et al., 2021b, Hastie and Tibshirani, 1995; Lin et al., 2019).

We assessed child stimulation in the home through family care indicator (FCI) score as a potential effect measure modifier, which is based on Home Observations for Measurement of the Environment (Hamadani et al., 2010). The FCI was previously found to be reliable for young children in Bangladesh (Hamadani et al., 2010). We assessed potential modification of the association between stress biomarkers and development outcome by FCI score at Year 1 for outcomes assessed at Years 1 and 2 and Year 2 for outcomes assessed at Year 2.

As these observational analyses were exploratory in nature, interpretations included both the strength of associations between individual biomarkers as well as the consistency of the direction of these associations across related biomarker groups. While typical corrections for false discovery rate aim to determine the probability of an individual result being due to random variation by adjusting for the number of repeated tests, we aimed to evaluate whether multiple measures of a similar exposure-outcome domain (e.g., salivary cortisol and child development) indicated an underlying association. For example, if we found that a domain of exposure-outcome associations (e.g. oxidative status and subsequent development) did not indicate a consistent direction of associations (i.e. some positive correlations and some negative correlations), but individual measures indicated statistically significant associations (e.g. iPF(2α)-VI at Year 1 and EASQ personal-social score at Year 2), we concluded that these individual results may be spurious associations due to repeated testing. On the other hand, if we found a consistent direction of associations (e.g., point estimates consistently indicating positive correlations) we concluded that these observed estimates might reflect a true association between the domain of exposures and outcomes. In addition, we corrected for repeated testing to evaluate the robustness of individual associations using the Benjamini-Hochberg procedure (Benjamini et al., 2001, Ferreira, 2007).

## 3 Ethics

Primary caregivers of all children provided written informed consent prior to enrollment. Human subjects protection committees at International Centre for Diarrhoeal Disease Research, Bangladesh (icddr,b), the University of California, Berkeley, and Stanford University approved the study protocols. Investigators registered the parent trial at ClinicalTrials.gov (NCT01590095) and a safety monitoring committee convened by icddr,b oversaw the study.

## 4 Results

**Fig. 1:**
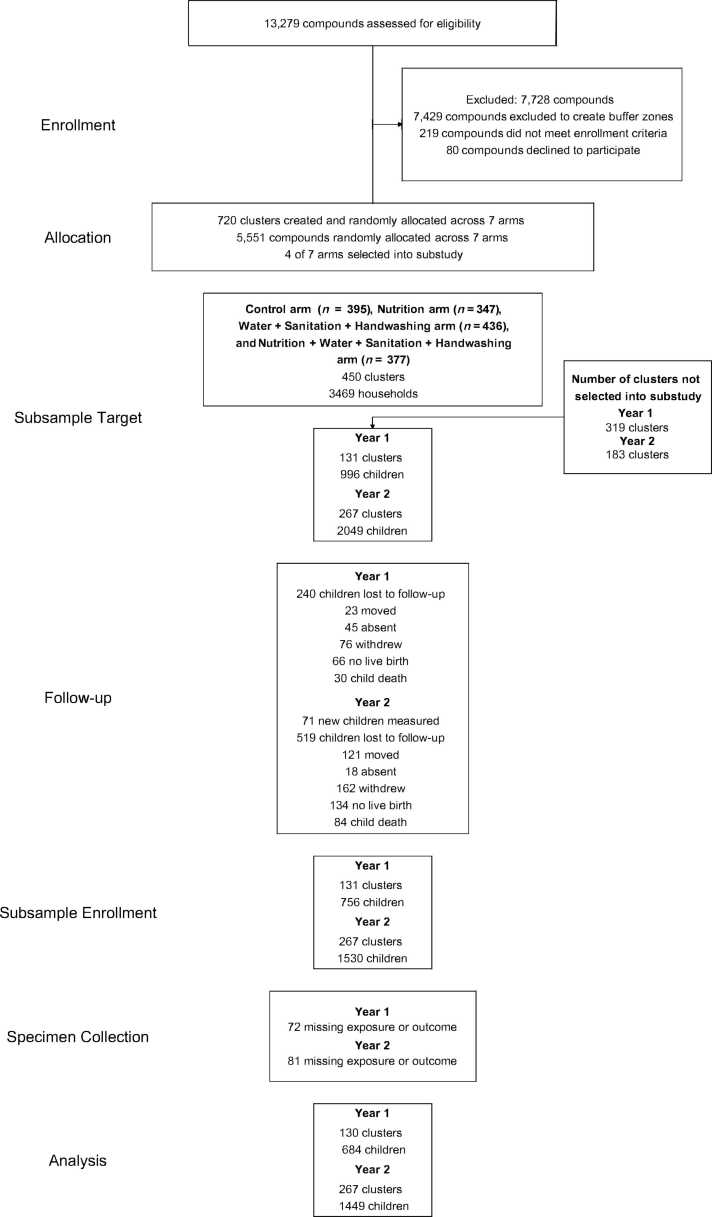
**Participant enrollment, follow-up, and analysis**.

We analyzed data from 684 children at Year 1 (median age 14 months) and 1449 children at Year 2 (median age 28 months) for this study (Fig. 1). The children had a median length-for-age z-score (LAZ) of −1.42 (IQR −2.07, −0.76) and a diarrhea prevalence of 13% at Year 1 and a median LAZ of −1.56 (IQR −2.28, −0.95) and a diarrhea prevalence of 7% at Year 2 (Table 1). Mothers of children in the sample had a median educational attainment of 7 years (IQR 4, 9) and a 57% prevalence of having experienced any type of intimate partner violence at some point in their lifetime. The children had a median cortisol reactivity of 0 (IQR 0.00, 0.01) μg/dl/min, median salivary alpha-amylase reactivity of 1.41 (IQR −0.08, 4.41) U/ml/min, median iPF(2α)-III of −0.42 (IQR −0.72, −0.09), median 2,3-dinor-iPF(2α)-III of 1.76 (IQR 1.55, 1.97), median iPF(2α)-VI of 2.57 (IQR 2.31, 2.87), median 8,12-iso-iPF(2α)-VI of 2.58 (IQR 2.17, 2.91), median mean arterial pressure 64.44 mmHg (IQR 60.78, 68.78), median resting heart rate 109 bpm (IQR 99.33, 118.67), median overall *NR3C1* exon 1F promoter methylation −5.66 (IQR −6.06, −5.32), and median methylation of NGFI-A transcription factor binding site −4.48 (IQR −4.65, −4.14) (Table 1). We evaluated the correlations between each stress biomarker and each development outcome (Supplemental Fig. 1). Overall, we found consistency between our adjusted and unadjusted estimates.

**Fig. 2:**
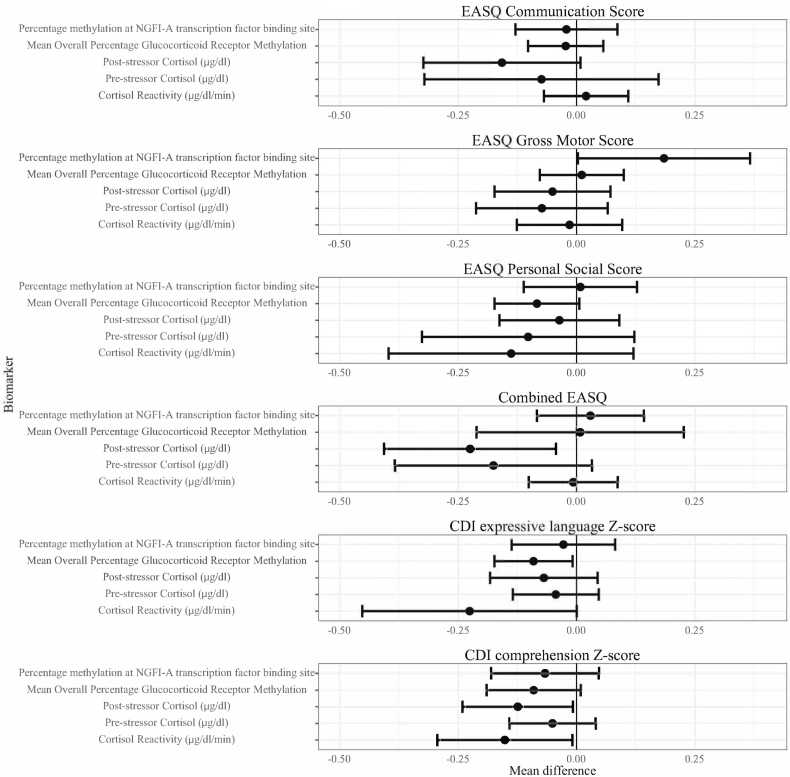
**HPA axis biomarkers and child development at Year 2.** HPA: Hypothalamic-pituitary-adrenal; CDI: MacArthur-Bates Communicative Development Inventories; EASQ: Extended Ages and Stages Questionnaire; sAA: salivary alpha-amylase. The difference in the mean child development outcome at the 75th and 25th percentile of each salivary stress biomarker exposure level and its 95% confidence interval after adjusting for relevant covariates.

**Fig. 3:**
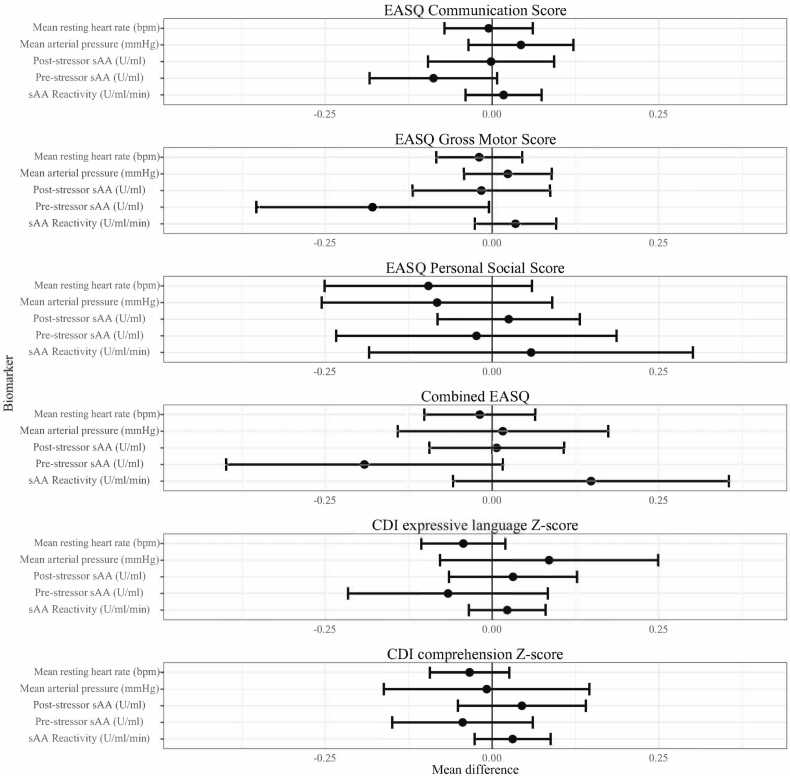
**SAM axis biomarkers and child development at Year 2.** SAM: Sympathetic-adrenal-medullary. CDI: MacArthur-Bates Communicative Development Inventories; EASQ: Extended Ages and Stages Questionnaire. The difference in the mean child development outcome at the 75th and 25th percentile of each measure of glucocorticoid receptor methylation and its 95% confidence interval after adjusting for relevant covariates.

**Fig. 4:**
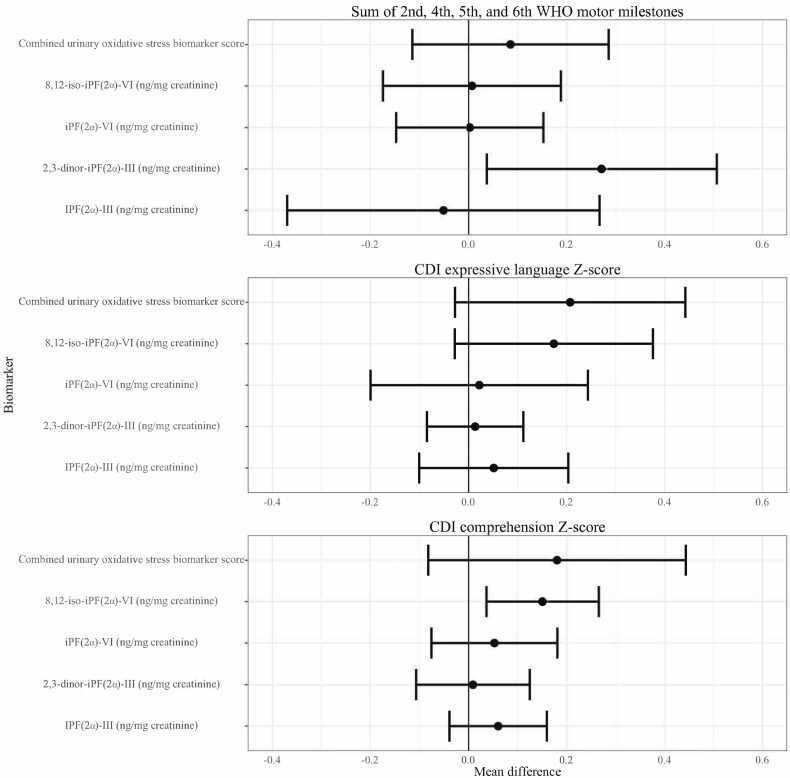
**Urinary isoprostanes and child development at Year 1.** CDI: MacArthur-Bates Communicative Development Inventories. The difference in the mean child development outcome at the 75th and 25th percentile of each measure of urinary isoprostanes and its 95% confidence interval after adjusting for relevant covariates.

**Fig. 5:**
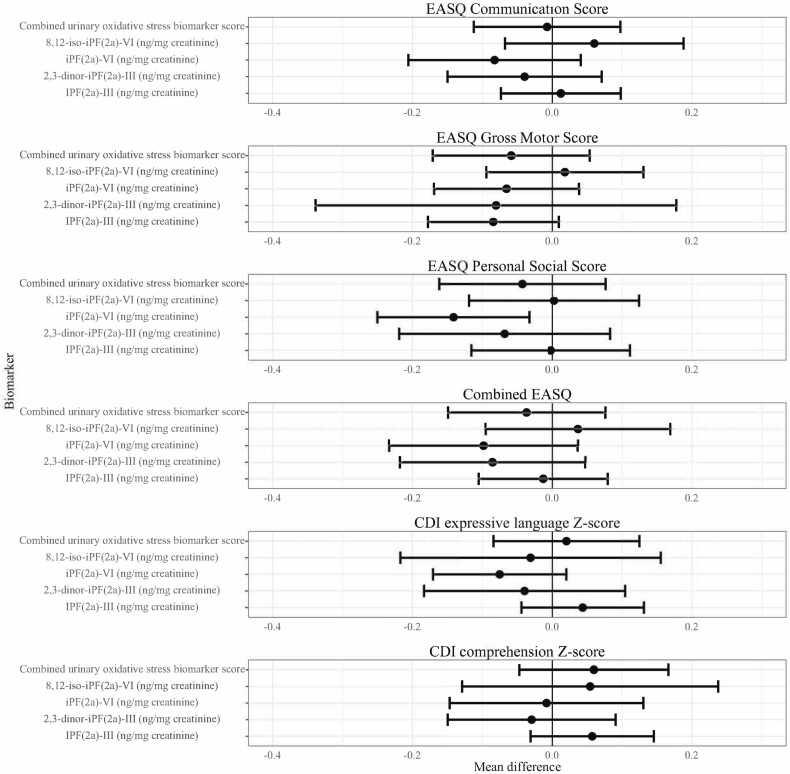
**Urinary isoprostanes at Year 1 and child development at Year 2.** CDI: MacArthur-Bates Communicative Development Inventories; EASQ: Extended Ages and Stages Questionnaire. The difference in the mean child development outcome at the 75th and 25th percentile of each measure of urinary isoprostanes and its 95% confidence interval after adjusting for relevant covariates.

We observed 135 associations (excluding subgroup analyses) of the differences in child development outcomes at the 75th and 25th percentiles of stress biomarkers across three domains of stress (HPA axis, SAM axis, and oxidative status). We found that markers of HPA axis activity (cortisol and glucocorticoid receptor methylation) were associated with child development in five out of 30 associations (17%; Fig. 2), markers of SAM activity (salivary alpha-amylase, heart rate, and blood pressure) were associated with child development in 1 out of 30 associations (3%; Fig. 3), and markers of oxidative status (F2-isoprostanes) were associated with child development in 3 out of 75 associations (4%, Fig. 4, Fig. 5). The proportion of significant results for HPA axis biomarkers was greater than we could expect due to random variation alone (5%; *α* = 0.05), but the proportion of significant results for SAM axis and oxidative status biomarkers were less than we would expect due to random variation, leading us to believe that significant associations with SAM axis and oxidative status biomarkers may be spurious due to repeated testing. No observed individual associations were statistically significant following false discovery rate correction for multiple testing.

### 4.1 HPA axis biomarkers

We assessed monotonicity of relationships by plotting the relationship (spline curves) between exposures and outcomes (Supplemental Fig. 3, Fig. 4). As spline curves may lead to erroneous inference based on inferences in the tails (i.e., outliers), we focused our inferences on values between the first and third quartiles. We generally found support for monotonicity in this region, which supports inference based on the difference between the 75th and 25th percentiles.

We found that increased salivary cortisol production and glucocorticoid receptor methylation were associated with worse child development outcomes (Fig. 2, Supplemental Table 2). These analyses indicated that increased concurrent cortisol reactivity was associated with a lower CDI comprehension score (adjusted difference −0.15 standard deviations (SD), 95% CI (−0.29, −0.01)) and a lower CDI expression score (adjusted difference −0.23 SD, 95% CI (−0.45, 0.00)) at Year 2. In addition, we found that higher post-stressor cortisol was associated with lower combined EASQ score (adjusted difference −0.22 SD, 95% CI (−0.41, −0.04)) and lower CDI comprehension score (adjusted difference −0.12 SD, 95% CI (−0.24, −0.01)). Greater mean overall glucocorticoid receptor methylation was correlated with lower concurrent CDI expressive language score (adjusted difference −0.09 SD, 95% CI (−0.17, −0.01)), and there was a consistently negative association between overall glucocorticoid receptor methylation and concurrent measures of child development (Fig. 2, Supplemental Table 2). A greater percent methylation of transcriptor NGFI-A binding site was associated with higher EASQ gross motor score (adjusted difference 0.18 SD, 95% CI (0.00, 0.37)), but this association was not consistent across other measures of child development. We found that cortisol reactivity was right-skewed, with the majority of children showing zero reactivity and a minority showing reactivity to the stressor (Supplemental Fig. 2).

### 4.2 SAM system biomarkers

We observed that greater pre-stressor salivary alpha-amylase was associated with worse child development outcomes (Fig. 3, Supplemental Table 3). There was a significant association between pre-stressor salivary alpha-amylase and EASQ gross motor score (adjusted difference −0.18 SD, 95% CI (-0.35, 0.00)), and the direction of this association was consistent across communication, personal social, and combined EASQ scores. We did not detect significant associations between post-stressor salivary alpha-amylase or salivary alpha-amylase reactivity and measures of development. Furthermore, we did not detect a significant association between mean arterial pressure or mean resting heart rate and any measures of concurrent development.

### 4.3 Oxidative status

We found limited evidence of an association between concurrent oxidative status and child development at Year 1 (Fig. 4, Supplemental Table 4). Increased concurrent 2,3-dinor-iPF(2α)-III (ng/mg creatinine) was associated with greater WHO motor milestones sum score (adjusted difference 0.27, 95% CI (0.04, 0.51)) as well as 8,12-iso-iPF(2α)-VI (ng/mg creatinine) and greater CDI comprehension Z-score (adjusted difference 0.15, 95% CI (0.04 SD, 0.27)). We assessed the possibility of a curvilinear association between concurrent oxidative status and child development by plotting the spline curves of these associations (Supplemental Fig. 4). The association between concurrent oxidative stress and CDI comprehension score largely indicated a positive correlation, while correlations of concurrent oxidative status and CDI expression and WHO sum score often depicted nonlinear associations, in which the second and third quartiles (moderate levels) of F2-isoprostanes were associated with better development outcomes relative to the first quartile.

We did not find evidence of a consistent association between measures of oxidative status at Year 1 and subsequent child development at Year 2 (Fig. 5, Supplemental Table 5). Higher levels of iPF(2α)-VI (ng/mg creatinine) were associated with lower EASQ personal-social score (adjusted difference −0.14, 95% CI (−0.25, −0.03)), but this inverse correlation was not consistent across other urinary F2-isoprostanes or measures of child development. We did not detect a significant association between any measure of oxidative status and time to attainment of any WHO motor milestone (Supplemental Table 6).

### 4.4 Modification by family care indicators

We analyzed potential modification of the association between stress biomarker and development outcome by family care indicators (FCI) score at Year 1 and Year 2. The contrasted FCI scores (75th and 25th percentiles) were 9 and 5 at Year 1, and 11 and 6 at Year 2. Although we found some evidence of effect measure modification in specific exposure-outcome associations at specific timepoints, these associations were not consistent over time (e.g., FCI at Year 1 and Year 2) or across related exposure-outcome domains (Supplemental Fig. 4).

## 5 Discussion

Our observations reveal some consistent associations between multi-level and multi-system biological signatures of exposure to chronic stress and early child development. Results suggest that unique biological responses to stress may be associated with the translation of experience and exposure into developmental consequences in early life. Yet, in this high risk (low-income, rural) developmental context in Bangladesh, using a large sample size, the magnitude of the effect was small to modest.

### 5.1 HPA axis

These findings indicate that HPA axis biomarkers, namely cortisol reactivity and overall glucocorticoid receptor methylation, are associated with developmental status, although the sensitivity of these measures is limited. This indicates that HPA axis activity may be a mechanism by which early-life adversity contributes to developmental impairment, although future studies such as mediation analyses should explore this hypothesis. While pre-stressor salivary alpha-amylase as well as moderate oxidative status showed some evidence of associations with developmental status, associations were most consistent and strongest for markers of HPA axis activity. These findings suggest that higher cortisol reactivity is associated with worse concurrent child development. This is consistent with previous studies’ findings that HPA axis hyperactivity may be related to delays in learning, memory, and neurological development (Booth et al., 2000, Franke, 2014, Grantham-McGregor et al., 2007, Lu et al., 2016, McEwen, 2011, National Scientific Council on the Developing Child, 2014). The majority of our sample demonstrated near-zero cortisol reactivity, which may indicate HPA axis dysregulation or that the stressor (venipuncture and separation form the mother) failed to elicit a stress response (e.g. due to social buffering via a secure attachment relationship), although we did not find evidence that this relationship was modified by stimulation in the home (FCI score) (Hostinar et al., 2014, Johnson et al., 2013, Reilly and Gunnar, 2019). Future studies should evaluate the drivers of HPA axis dysregulation in this sample in order to further elucidate these pathways of interest.

The age of the sample population has considerable implications, as early childhood is a particularly sensitive period to stress (Walker et al., 2011). In school-aged children, low or blunted (little change throughout the day) cortisol production is associated with poor developmental outcomes, and investigators have hypothesized that HPA axis hypoactivity through childhood and adulthood may be the result of early-life HPA axis hyperactivity (Gunnar and Vazquez, 2001). Follow-up evaluation of HPA axis activity in this cohort once they have reached school age may provide insights on the longer-term developmental correlates of early-life HPA axis activity.

We found that greater glucocorticoid receptor methylation was associated with worse child development outcomes. This indicates that glucocorticoid receptor hypermethylation, which is an indicator of early-life stress, may be associated with poor developmental outcomes for high-risk children in rural Bangladesh (van der Knaap et al., 2014). This is consistent with previous findings that glucocorticoid receptor methylation is positively associated with externalizing behavior and depressive symptoms in school-aged children (Cicchetti and Handley, 2017). In a previous analysis of this sample evaluating the impact of randomized assignment to interventions on child stress, we found that the control group had greater glucocorticoid receptor methylation relative to the combined nutrition, water, sanitation, and hygiene intervention group (Lin et al., 2021c). These cumulative findings indicate that glucocorticoid receptor methylation may be a pathway or marker of environmental stressors’ contribution to developmental status, but this hypothesis should be investigated further by future studies.

Multiple studies in animal and human models have found that early life exposure to glucocorticoids can lead to long-term dysregulation of the HPA axis and cascading consequences across the epigenetic landscape (Dantzer, 2023, Lee and Sawa, 2014, Majer et al., 2023). While the biological mechanism of this relationship is unclear, glucocorticoid disruption can alter brain-derived neurotrophic factor and tyrosine hydroxylase genes, which in turn can affect behavior and development (Lee and Sawa, 2014). Furthermore, excess glucocorticoids can have toxic effects on the hippocampus, which is rich with glucocorticoid receptors and regulates learning and memory (Finsterwald and Alberini, 2014, Oakley et al., 2021). This sample of low-income, rural children with a high burden of environmental stressors, provides a unique perspective on the role of the HPA axis as it relates to child development. While much of the literature related to markers of stress in the HPA axis in humans has focused on psychosocial stressors, which are often buffered by a secure attachment relationship in infancy and early childhood, these findings provide additional support regarding the importance of the HPA axis response as it pertains to child development for children facing a high burden of environmental stressors (Rana et al., 2022, Walker et al., 2011, Walker et al., 2007). In particular, our findings support that the impact of environmental stressors on child development may be mediated by glucocorticoids and the HPA axis, rather than the SAM system or oxidative status. Future studies should further explore this relationship by evaluating potential mediation of the relationship between environmental stressors (e.g. pathogen exposure, poverty, malnutrition) and developmental status by HPA axis biomarkers.

### 5.2 SAM axis and oxidative status

Although we detected a positive correlation between pre-stressor salivary alpha-amylase and child development, the lack of consistency of relationships (in terms of direction and significance) across measures of SAM axis activity and child development (3% of associations were significant) indicates that this association may be spurious. Similarly, although we found limited evidence that moderate oxidative status was associated with child development, the inconsistency of these correlations across measures of oxidative status and child development (4% of associations were significant) are quite consistent with random variation.

### 5.3 Strengths and limitations

A major strength of this study is that it included a large cohort of children from the global majority population with well-documented health information from gestation to early childhood. Our study evaluated the association between stress and child development using a comprehensive set of biomarkers representing the HPA axis, SAM system, and oxidative status. As each of these biomarkers reflects a unique stress response, analysis of these individual correlations between each stress biomarker and measure of child development allows for evaluation of these associations at multiple levels and multiple biological systems.

The majority of the analyses conducted were with concurrent exposure and outcome data, which does not readily enable causal inference regarding the impact of child stress on child development. We conducted observational analyses with multivariate adjustment to control for potential confounders and covariates of interest, although residual confounding may still be present. Future analyses should include a greater number of time points for observations with additional temporal separation. Our interpretations for concurrent analyses are based on the assumption that stress biomarker exposures may cause a change in child development, although it is possible that child development outcomes lead to changes in child stress neurobiology (i.e., reverse causation). In addition, assessment of child development measures for children greater than 3 years of age, such as school attendance, executive functioning, and intelligence, would enable inference of the impact of early stress on longer-term development.

The inclusion of multiple measures of both stress and development is both a strength of this study and a limitation, as multiple comparisons lead to an increased risk of Type 1 error. We aimed to account for this risk by assessing the consistency of the direction (positive vs. negative) of point estimates in each domain of exposure-outcome assessments, in addition to evaluation of each contrast’s statistical significance, although the possibility of Type 1 errors remains plausible. Furthermore, the use of corrections for false discovery, such as Benjamini-Hochberg, may be overly conservative (i.e., low power) for correlational studies of stress biomarkers and child development, where we would expect to see small to modest effect sizes. Therefore, we recommend that these inferences inform futures studies that can deliberately target and evaluate potential associations of interest.

## 6 Conclusions

Our findings indicate a relationship between HPA axis biomarkers (cortisol and glucocorticoid receptor methylation) and developmental status of young children in Bangladesh. This indicates that HPA axis biomarkers may represent a mechanistic pathway by which early life stressors lead to subsequent development, although this hypothesis should be investigated by future studies such as mediation analyses. These associations contribute to the body of evidence that supports interventions that aim to improve child development by intervening on early-life stress, such as family-based interventions that target multiple stressors (Fisher, 2016, Pitchik et al., 2021).

## CRediT authorship contribution statement

**Mahbubur Rahman:** Writing – review & editing. **Leanne Unicomb:** Writing – review & editing. **Md. Saheen Hossen:** Writing – review & editing. **Palash Mutsuddi:** Writing – review & editing. **Ann Meyer:** Writing – review & editing. **Andrew N. Mertens:** Conceptualization, Formal analysis, Supervision, Writing – review & editing. **Christine P. Stewart:** Writing – review & editing. **Sophia T. Tan:** Formal analysis, Writing – review & editing, Visualization. **Kishor K. Das:** Writing – review & editing. **Liying Yan:** Writing – review & editing. **Zachary Butzin-Dozier:** Formal analysis, Writing – original draft, Writing – review & editing. **Douglas A. Granger:** Conceptualization, Supervision, Writing – review & editing. **Helen O. Pitchik:** Writing – review & editing. **Abul K. Shoab:** Writing – review & editing. **Salma Akther:** Writing – review & editing. **John M. Colford Jr.:** Writing – review & editing. **Ivan Spasojevic:** Writing – review & editing. **Lia C. H. Fernald:** Conceptualization, Supervision, Writing – review & editing. **Idan Shalev:** Writing – review & editing. **Md. Mahfuz Al Mamun:** Writing – review & editing. **Fahmida Tofail:** Writing – review & editing. **Stephen P. Luby:** Writing – review & editing. **Md. Ziaur Rahman:** Writing – review & editing. **Sunny Shahriar:** Writing – review & editing. **Syeda Luthfa Famida:** Writing – review & editing. **Audrie Lin:** Conceptualization, Data curation, Funding acquisition, Investigation, Project administration, Resources, Supervision, Writing – review & editing. **Shahjahan Ali:** Writing – review & editing. **Mohammed Rabiul Karim:** Writing – review & editing. **Gabrielle Shuman:** Data curation, Writing – review & editing. **Ruchira Tabassum Naved:** Writing – review & editing. **Kausar Parvin:** Writing – review & editing. **Dora Il'yasova:** Writing – review & editing. **Alan E. Hubbard:** Writing – review & editing.

## Declaration of Competing Interest

This work was supported by Global Development grants [OPPGD759 and OPP1165144] from the Bill & Melinda Gates Foundation to the University of California, Berkeley and by the National Institute of Allergy and Infectious Diseases of the National Institutes of Health [grant number K01AI136885 to AL]. The content is solely the responsibility of the authors and does not necessarily represent the official views of the National Institutes of Health. In the interest of full disclosure and transparency, we would like to disclose the roles of co-authors. Douglas Granger is the founder and chief scientific and strategy advisor at Salimetrics LLC and SalivaBio LLC, and these relationships are managed by the policies of the committees on conflict of interest at the Johns Hopkins University School of Medicine and the University of California at Irvine. Liying Yan is the president of EpigenDx, Inc. Ann Meyer is the Associate Director of Operations at EpigenDx, Inc.

## References

[bib1] Afifi T., Boman J., Fleisher W., Sareen J. (2009). The relationship between child abuse, parental divorce, and lifetime mental disorders and suicidality in a nationally representative adult sample. Child Abus. Negl..

[bib2] Amstadter A.B., Moscati A., Maes H.H., Myers J.M., Kendler K.S. (2016). Personality, cognitive/psychological traits and psychiatric resilience: a multivariate twin study. Personal. Individ. Differ..

[bib3] Aschbacher K., O’Donovan A., Wolkowitz O.M., Dhabhar F.S., Su Y., Epel E. (2013). Good stress, bad stress and oxidative stress: insights from anticipatory cortisol reactivity. Psychoneuroendocrinology.

[bib4] Asok A., Bernard K., Roth T.L., Rosen J.B., Dozier M. (2013). Parental responsiveness moderates the association between early-life stress and reduced telomere length. Dev. Psychopathol..

[bib5] Benjamini Y., Drai D., Elmer G., Kafkafi N., Golani I. (2001). Controlling the false discovery rate in behavior genetics research. Behav. Brain Res..

[bib6] Bernard K., Dozier M., Bick J., Gordon M.K. (2015). Intervening to enhance cortisol regulation among children at risk for neglect: Results of a randomized clinical trial. Dev. Psychopathol..

[bib7] Black M.M., Walker S.P., Fernald L.C.H., Andersen C.T., DiGirolamo A.M., Lu C., McCoy D.C., Fink G., Shawar Y.R., Shiffman J., Devercelli A.E., Wodon Q.T., Vargas-Baron E., Grantham-McGregor S. (2017). Early childhood development coming of age: science through the life course. Lancet.

[bib8] Booth A., Carver K., Granger D.A. (2000). Biosocial perspectives on the family. J. Marriage Fam..

[bib9] Bourke C.D., Berkley J.A., Prendergast A.J. (2016). Immune dysfunction as a cause and consequence of malnutrition. Trends Immunol..

[bib10] Brietzke E., Kauer Sant’anna M., Jackowski A., Grassi-Oliveira R., Bucker J., Zugman A., Mansur R.B., Bressan R.A. (2012). Impact of childhood stress on psychopathology. Braz. J. Psychiatry.

[bib11] Chu, B., Marwaha, K., Sanvictores, T., Ayers, D., 2023. Physiology, Stress Reaction., in: StatPearls. StatPearls Publishing, Treasure Island (FL). 31082164

[bib12] Cicchetti D., Handley E.D. (2017). Methylation of the glucocorticoid receptor gene, nuclear receptor subfamily 3, group C, member 1 (NR3C1), in maltreated and nonmaltreated children: Associations with behavioral undercontrol, emotional lability/negativity, and externalizing and internalizing symptoms. Dev. Psychopathol..

[bib13] Coe C.L., Kramer M., Czéh B., Gould E., Reeves A.J., Kirschbaum C., Fuchs E. (2003). Prenatal stress diminishes neurogenesis in the dentate gyrus of juvenile rhesus monkeys. Biol. Psychiatry.

[bib14] Condon E.M. (2018). Chronic stress in children and adolescents: a review of biomarkers for use in pediatric research. Biol. Res. Nurs..

[bib15] Curtis M.J., Bond R.A., Spina D., Ahluwalia A., Alexander S.P.A., Giembycz M.A., Gilchrist A., Hoyer D., Insel P.A., Izzo A.A., Lawrence A.J., MacEwan D.J., Moon L.D.F., Wonnacott S., Weston A.H., McGrath J.C. (2015). Experimental design and analysis and their reporting: new guidance for publication in BJP. Br. J. Pharmacol..

[bib16] Dantzer B. (2023). Frank Beach Award Winner: the centrality of the hypothalamic-pituitary-adrenal axis in dealing with environmental change across temporal scales. Horm. Behav..

[bib17] Del Giudice M., Buck C.L., Chaby L.E., Gormally B.M., Taff C.C., Thawley C.J., Vitousek M.N., Wada H. (2018). What is stress? A systems perspective. Integr. Comp. Biol. 58, 1019–1032.

[bib18] van der Knaap L.J., Riese H., Hudziak J.J., Verbiest M.M.P.J., Verhulst F.C., Oldehinkel A.J., van Oort F.V.A. (2014). Glucocorticoid receptor gene (NR3C1) methylation following stressful events between birth and adolescence. The TRAILS study. Transl. Psychiatry.

[bib19] Edelman S., Shalev I., Uzefovsky F., Israel S., Knafo A., Kremer I., Mankuta D., Kaitz M., Ebstein R.P. (2012). Epigenetic and genetic factors predict women’s salivary cortisol following a threat to the social self. PLoS One.

[bib20] Engle P.L., Black M.M., Behrman J.R., Cabral de Mello M., Gertler P.J., Kapiriri L., Martorell R., Young M.E. (2007). Strategies to avoid the loss of developmental potential in more than 200 million children in the developing world. Lancet.

[bib21] Engle P.L., Fernald L.C.H., Alderman H., Behrman J., O’Gara C., Yousafzai A., de Mello M.C., Hidrobo M., Ulkuer N., Ertem I., Iltus S. (2011). Strategies for reducing inequalities and improving developmental outcomes for young children in low-income and middle-income countries. Lancet.

[bib22] Fagan T.C., Conrad K.A., Mayshar P.V., Mackie M.J., Hagaman R.M. (1988). Single versus triplicate measurements of blood pressure and heart rate. Hypertension.

[bib23] Fernald L.C., Grantham-McGregor S.M. (1998). Stress response in school-age children who have been growth retarded since early childhood. Am. J. Clin. Nutr..

[bib24] Fernald L.C., Grantham-McGregor S.M., Manandhar D.S., Costello A. (2003). Salivary cortisol and heart rate in stunted and nonstunted Nepalese school children. Eur. J. Clin. Nutr..

[bib25] Fernald L.C.H., Gunnar M.R. (2009). Poverty-alleviation program participation and salivary cortisol in very low-income children. Soc. Sci. Med..

[bib26] Fernald L.C.H., Kariger P., Hidrobo M., Gertler P.J. (2012). Socioeconomic gradients in child development in very young children: evidence from India, Indonesia, Peru, and Senegal. Proc. Natl. Acad. Sci. USA.

[bib27] Ferreira J.A. (2007). The Benjamini-Hochberg method in the case of discrete test statistics. Int J. Biostat..

[bib28] Finsterwald C., Alberini C.M. (2014). Stress and glucocorticoid receptor-dependent mechanisms in long-term memory: from adaptive responses to psychopathologies. Neurobiol. Learn Mem..

[bib29] Fisher P.A. (2016). Translational neuroscience as a tool for intervention development in the context of high-adversity families. N. Dir. Child Adolesc. Dev..

[bib30] Fisher P.A., Stoolmiller M., Gunnar M.R., Burraston B.O. (2007). Effects of a therapeutic intervention for foster preschoolers on diurnal cortisol activity. Psychoneuroendocrinology.

[bib31] Franke H.A. (2014). Toxic stress: effects, prevention and treatment. Children.

[bib32] Godoy L.D., Rossignoli M.T., Delfino-Pereira P., Garcia-Cairasco N., de Lima Umeoka E.H. (2018). A comprehensive overview on stress neurobiology: basic concepts and clinical implications. Front. Behav. Neurosci..

[bib33] Granger D.A., Kivlighan K.T., el-Sheikh M., Gordis E.B., Stroud L.R. (2007). Salivary alpha-amylase in biobehavioral research: recent developments and applications. Ann. N. Y Acad. Sci..

[bib34] Granger Douglas A., Kivlighan K.T., Fortunato C., Harmon A.G., Hibel L.C., Schwartz E.B., Whembolua G.-L. (2007). Integration of salivary biomarkers into developmental and behaviorally-oriented research: problems and solutions for collecting specimens. Physiol. Behav..

[bib35] Grantham-McGregor S., Cheung Y.B., Cueto S., Glewwe P., Richter L., Strupp B. (2007). Developmental potential in the first 5 years for children in developing countries. Lancet.

[bib36] Gunnar M.R., Fisher P.A. (2006). Bringing basic research on early experience and stress neurobiology to bear on preventive interventions for neglected and maltreated children. Dev. Psychopathol..

[bib37] Gunnar M.R., Vazquez D.M. (2001). Low cortisol and a flattening of expected daytime rhythm: potential indices of risk in human development. Dev. Psychopathol..

[bib38] Hamadani Jena D., Tofail F., Hilaly A., Huda S.N., Engle P., Grantham-McGregor S.M. (2010). Use of family care indicators and their relationship with child development in Bangladesh. J. Health Popul Nutr..

[bib39] Hastie T., Tibshirani R. (1995). Generalized additive models for medical research. Stat. Methods Med. Res..

[bib40] Hostinar C.E., Sullivan R.M., Gunnar M.R. (2014). Psychobiological mechanisms underlying the social buffering of the hypothalamic–pituitary–adrenocortical axis: A review of animal models and human studies across development. Psychol. Bull..

[bib41] Huth-Bocks A.C., Levendosky A.A., Bogat G.A. (2002). The effects of domestic violence during pregnancy on maternal and infant health. Violence Vict..

[bib42] Il’yasova D., Spasojevic I., Wang F., Tolun A.A., Base K., Young S.P., Marcom P.K., Marks J., Mixon G., DiGiulio R., Millington D.S. (2010). Urinary biomarkers of oxidative status in a clinical model of oxidative assault. Cancer Epidemiol. Biomark. Prev..

[bib43] Jansen J., Beijers R., Riksen-Walraven M., de Weerth C. (2010). Cortisol reactivity in young infants. Psychoneuroendocrinology.

[bib44] Jiang S., Postovit L., Cattaneo A., Binder E.B., Aitchison K.J. (2019). Epigenetic modifications in stress response genes associated with childhood trauma. Front Psychiatry.

[bib45] Johnson S.B., Riley A.W., Granger D.A., Riis J. (2013). The science of early life toxic stress for pediatric practice and advocacy. Pediatrics.

[bib46] Jolliffe I.T., Cadima J. (2016). Principal component analysis: a review and recent developments. Philos. Trans. A Math. Phys. Eng. Sci..

[bib47] Kagan J., Reznick J.S., Snidman N. (1987). The physiology and psychology of behavioral inhibition in children. Child Dev..

[bib48] Labella M.H., Eiden R.D., Tabachnick A.R., Sellers T., Dozier M. (2021). Infant neurodevelopmental outcomes of prenatal opioid exposure and polysubstance use. Neurotoxicol Teratol..

[bib49] Lee R.S., Sawa A. (2014). Environmental stressors and epigenetic control of the hypothalamic-pituitary-adrenal axis. Neuroendocrinology.

[bib50] Levendosky A.A., Bogat G.A., Martinez-Torteya C. (2013). PTSD symptoms in young children exposed to intimate partner violence. Violence Women.

[bib51] Lin A., Ali S., Arnold B.F., Rahman M.Z., Alauddin M., Grembi J., Mertens A.N., Famida S.L., Akther S., Hossen M.S., Mutsuddi P., Shoab A.K., Hussain Z., Rahman M., Unicomb L., Ashraf S., Naser A.M., Parvez S.M., Ercumen A., Benjamin-Chung J., Haque R., Ahmed T., Hossain M.I., Choudhury N., Jannat K., Alauddin S.T., Minchala S.G., Cekovic R., Hubbard A.E., Stewart C.P., Dewey K.G., Colford J.M., Luby S.P. (2020). Effects of Water, Sanitation, Handwashing, and Nutritional Interventions on Environmental Enteric Dysfunction in Young Children: A Cluster-randomized, Controlled Trial in Rural Bangladesh. Clin. Infect. Dis..

[bib52] Lin A., Mertens A.N., Arnold B.F., Tan S., Lin J., Stewart C.P., Hubbard A.E., Ali S., Benjamin-Chung J., Shoab A.K., Rahman M.Z., Famida S.L., Hossen M.S., Mutsuddi P., Akther S., Rahman M., Unicomb L., Naved R.T., Mamun M.M.A., Parvin K., Dhabhar F.S., Kariger P., Fernald L.C., Luby S.P., Colford J.M. (2021). Telomere length is associated with growth in children in rural Bangladesh. Elife.

[bib53] Lin, A., Mertens, A., Li, A., Butzin-Dozier, Z., Tan, S., 2021a. WASH Benefits Bangladesh Child Stress and Child Development Analysis Plan.

[bib54] Lin, A., Mertens, A.N., Rahman, Md.Z., Tan, S., Il’yasova, D., Spasojevic, I., Ali, S., Stewart, C.P., Fernald, L.C.H., Kim, L., Yan, L., Meyer, A., Karim, Md.R., Shahriar, S., Shuman, G., Arnold, B.F., Hubbard, A., Famida, S.L., Akther, S., Hossen, Md.S., Mutsuddi, P., Shoab, A.K., Shalev, I., Rahman, M., Unicomb, L., Heaney, C.D., Kariger, P., Colford, J.M., Luby, S.P., Granger, D.A., 2021c. Effects of drinking water, sanitation, handwashing and nutritional interventions on stress physiology, oxidative stress, and epigenetic programming in young children living in rural Bangladesh: A randomized clinical trial.

[bib55] Lin F.A.N., Qing G.U., Qiang Z.E.N.G. (2019). Progress in the application of generalized additive model in epidemiologic studies on air pollution. J. Environ. Occup. Med..

[bib56] Loman M.M., Gunnar M.R., Early Experience S., Neurobehavioral development center (2010). Early experience and the development of stress reactivity and regulation in children. Neurosci. Biobehav Rev..

[bib57] Lu C., Black M.M., Richter L.M. (2016). Risk of poor development in young children in low-income and middle-income countries: an estimation and analysis at the global, regional, and country level. Lancet Glob. Health.

[bib58] Luby S.P., Rahman M., Arnold B.F., Unicomb L., Ashraf S., Winch P.J., Stewart C.P., Begum F., Hussain F., Benjamin-Chung J., Leontsini E., Naser A.M., Parvez S.M., Hubbard A.E., Lin A., Nizame F.A., Jannat K., Ercumen A., Ram P.K., Das K.K., Abedin J., Clasen T.F., Dewey K.G., Fernald L.C., Null C., Ahmed T., Colford J.M. (2018). Effects of water quality, sanitation, handwashing, and nutritional interventions on diarrhoea and child growth in rural Bangladesh: a cluster randomised controlled trial. Lancet Glob. Health.

[bib59] Majer A.D., Paitz R.T., Tricola G.M., Geduldig J.E., Litwa H.P., Farmer J.L., Prevelige B.R., McMahon E.K., McNeely T., Sisson Z.R., Frenz B.J., Ziur A.D., Clay E.J., Eames B.D., McCollum S.E., Haussmann M.F. (2023). The response to stressors in adulthood depends on the interaction between prenatal exposure to glucocorticoids and environmental context. Sci. Rep..

[bib60] McEwen B.S. (2000). Allostasis and allostatic load: implications for neuropsychopharmacology. Neuropsychopharmacology.

[bib61] McEwen B.S. (2011). Effects of stress on the developing brain. Cerebrum.

[bib62] Montuschi P., Barnes P.J., Roberts L.J. (2004). Isoprostanes: markers and mediators of oxidative stress. FASEB J..

[bib63] Nater U.M., Rohleder N. (2009). Salivary alpha-amylase as a non-invasive biomarker for the sympathetic nervous system: current state of research. Psychoneuroendocrinology.

[bib64] National Scientific Council on the Developing Child, 2014. Excessive Stress Disrupts the Architecture of the Developing Brain (No. 3).

[bib65] Ndayizigiye M., McBain R., Whelley C., Lerotholi R., Mabathoana J., Carmona M., Curtain J., Birru E., Stulac S., Miller A.C., Shin S., Rumaldo N., Mukherjee J., Nelson A.K. (2022). Integrating an early child development intervention into an existing primary healthcare platform in rural Lesotho: a prospective case-control study. BMJ Open.

[bib66] Nychka D. (1988). Bayesian Confidence Intervals for Smoothing Splines. J. Am. Stat. Assoc..

[bib67] Oakley R.H., Whirledge S.D., Petrillo M.G., Riddick N.V., Xu X., Moy S.S., Cidlowski J.A. (2021). Combinatorial actions of glucocorticoid and mineralocorticoid stress hormone receptors are required for preventing neurodegeneration of the mouse hippocampus. Neurobiol. Stress.

[bib68] Pitchik H.O., Tofail F., Rahman M., Akter F., Sultana J., Shoab A.K., Huda T.Md.N., Jahir T., Amin M.R., Hossain M.K., Das J.B., Chung E.O., Byrd K.A., Yeasmin F., Kwong L.H., Forsyth J.E., Mridha M.K., Winch P.J., Luby S.P., Fernald L.C. (2021). A holistic approach to promoting early child development: a cluster randomised trial of a group-based, multicomponent intervention in rural Bangladesh. BMJ Glob. Health.

[bib69] Pizzino G., Irrera N., Cucinotta M., Pallio G., Mannino F., Arcoraci V., Squadrito F., Altavilla D., Bitto A. (2017). Oxidative Stress: Harms and Benefits for Human Health. Oxid. Med Cell Longev..

[bib70] Rana J., Luna-Gutiérrez P., Haque S.E., Ignacio Nazif-Muñoz J., Mitra D.K., Oulhote Y. (2022). Associations between household air pollution and early child development among children aged 36–59 months in Bangladesh. J. Epidemiol. Community Health.

[bib71] Rehman, S., Nelson, V.L., 2022. Blood Pressure Measurement., in: StatPearls. StatPearls Publishing, Treasure Island (FL). 29489154

[bib72] Reilly E.B., Gunnar M.R. (2019). Neglect, HPA axis reactivity, and development. Int J. Dev. Neurosci..

[bib73] Schmidt K.L., Merrill S.M., Gill R., Miller G.E., Gadermann A.M., Kobor M.S. (2021). Society to cell: How child poverty gets “Under the Skin” to influence child development and lifelong health. Dev. Rev..

[bib74] Shonkoff J.P., Boyce W.T., Bush N.R., Gunnar M.R., Hensch T.K., Levitt P., Meaney M.J., Nelson C.A., Slopen N., Williams D.R., Silveira P.P. (2022). Translating the Biology of Adversity and Resilience Into New Measures for Pediatric Practice. Pediatrics.

[bib75] Singh A., Yeh C.J., Boone Blanchard S. (2017). Ages and stages questionnaire: a global screening scale. Bol. Med Hosp. Infant Mex..

[bib76] Tofail F., Fernald L.C., Das K.K., Rahman M., Ahmed T., Jannat K.K., Unicomb L., Arnold B.F., Ashraf S., Winch P.J., Kariger P., Stewart C.P., Colford J.M., Luby S.P. (2018). Effect of water quality, sanitation, hand washing, and nutritional interventions on child development in rural Bangladesh (WASH Benefits Bangladesh): a cluster-randomised controlled trial. Lancet Child Adolesc. Health.

[bib77] Walker S.P., Wachs T.D., Meeks Gardner J., Lozoff B., Wasserman G.A., Pollitt E., Carter J.A. (2007). Child development: risk factors for adverse outcomes in developing countries. Lancet.

[bib78] Walker S.P., Wachs T.D., Grantham-McGregor S., Black M.M., Nelson C.A., Huffman S.L., Baker-Henningham H., Chang S.M., Hamadani J.D., Lozoff B., Gardner J.M.M., Powell C.A., Rahman A., Richter L. (2011). Inequality in early childhood: risk and protective factors for early child development. Lancet.

[bib79] WHO Motor Development Study: windows of achievement for six gross motor development milestones., 2006. Acta Paediatr Suppl 450, 86–95. 10.1111/j.1651-2227.2006.tb02379.x. 16817682

[bib80] Wolf E.J., Miller M.W., Sullivan D.R., Amstadter A.B., Mitchell K.S., Goldberg J., Magruder K.M. (2018). A classical twin study of PTSD symptoms and resilience: Evidence for a single spectrum of vulnerability to traumatic stress. Depress Anxiety.

[bib81] Wood S.N., Pya N., Säfken B. (2016). Smoothing Parameter and Model Selection for General Smooth Models. J. Am. Stat. Assoc..

[bib82] Yaribeygi H., Panahi Y., Sahraei H., Johnston T.P., Sahebkar A. (2017). The impact of stress on body function: A review. EXCLI J..

[bib83] Zhang T.Y., Labonté B., Wen X.L., Turecki G., Meaney M.J. (2013). Epigenetic mechanisms for the early environmental regulation of hippocampal glucocorticoid receptor gene expression in rodents and humans. Neuropsychopharmacology.

