# Supplementary material for: Stress biomarkers and child development in young children in Bangladesh
Source: Psychoneuroendocrinology. 2024 Jun;164:107023. doi: 10.1016/j.psyneuen.2024.107023 (PMC11157411; doi:10.1016/j.psyneuen.2024.107023)
Supplement: Supplementary file 1 — Supplementary material [file mmc1.pdf]

Supplemental Materials

Appendix 1. Analysis methods and covariates

We assessed the following baseline enrollment covariates – child birth order, maternal age, maternal height, maternal education, household food insecurity, number of children in the household, number of individuals living in the compound, distance to primary drinking water source, and a household asset score calculated via the first principal component of a principal components analysis. In addition, based on literature review and subject area knowledge, we identified time-varying covariates that were possibly associated with the outcomes of interest but were not likely to be caused by either the exposure or outcome of interest. These time-varying covariates included – prior length-for-age Z-score (LAZ, categorized), prior weight-for-age Z-score (WAZ, categorized), month of measurement, treatment arm (control, N+WSH), pre-stressor sample collection time, maternal Center for Epidemiologic Studies Depression Revised (CESD-R) Scale score,[11] maternal Perceived Stress Scale (PSS) score,[12] and maternal lifetime cumulative exposure to intimate partner violence as measured by the WHO Women's Health and Life Experiences Survey.[13] Additional information regarding the rationale for covariate inclusion and temporality of assessment can be found in this study's pre-registered analysis plan.[14]

Supplemental Figure 1. Pearson’s correlation between stress biomarkers and child development outcomes

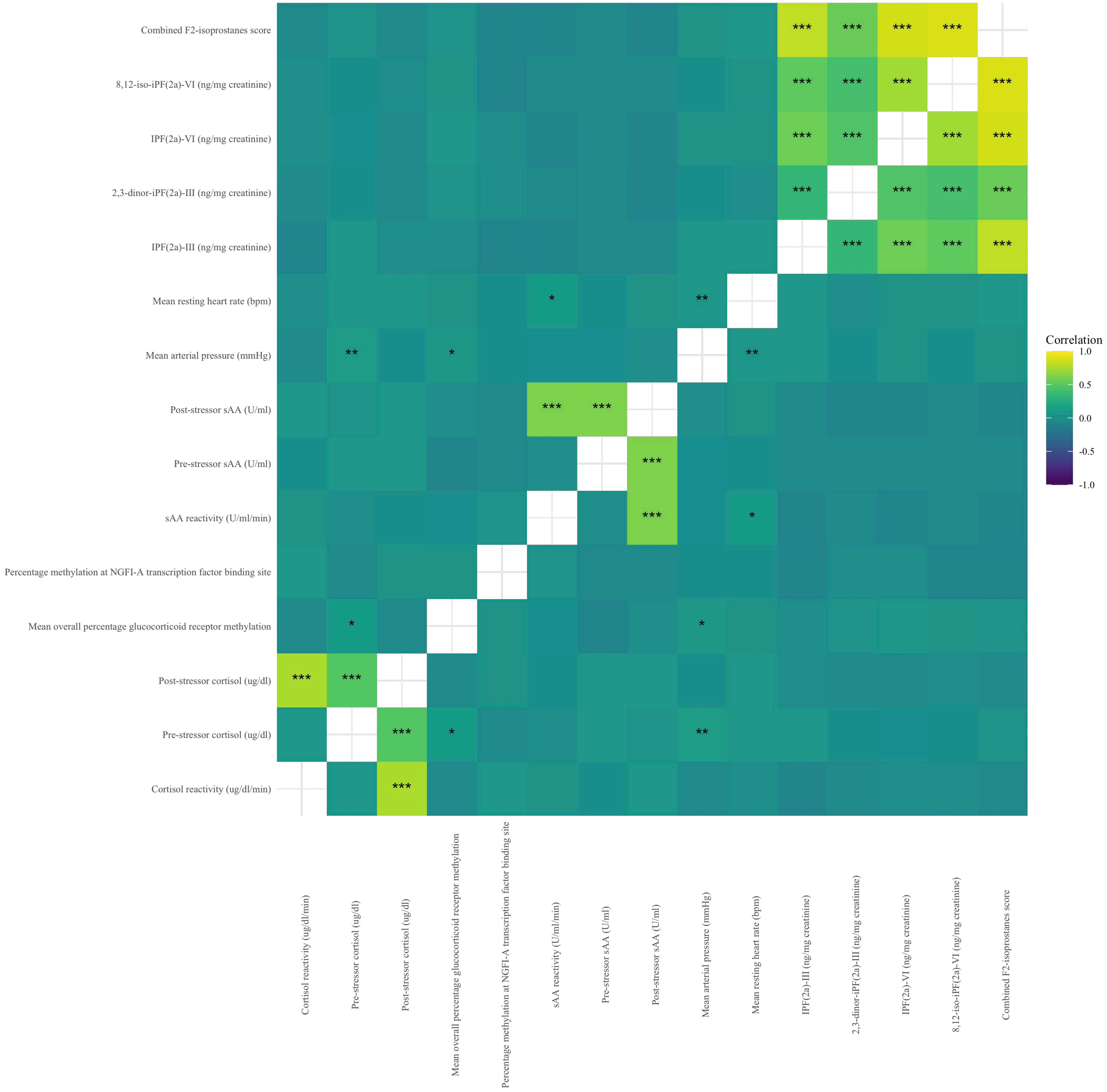

Supplemental Figure 2. Distribution of child cortisol reactivity at Year 2

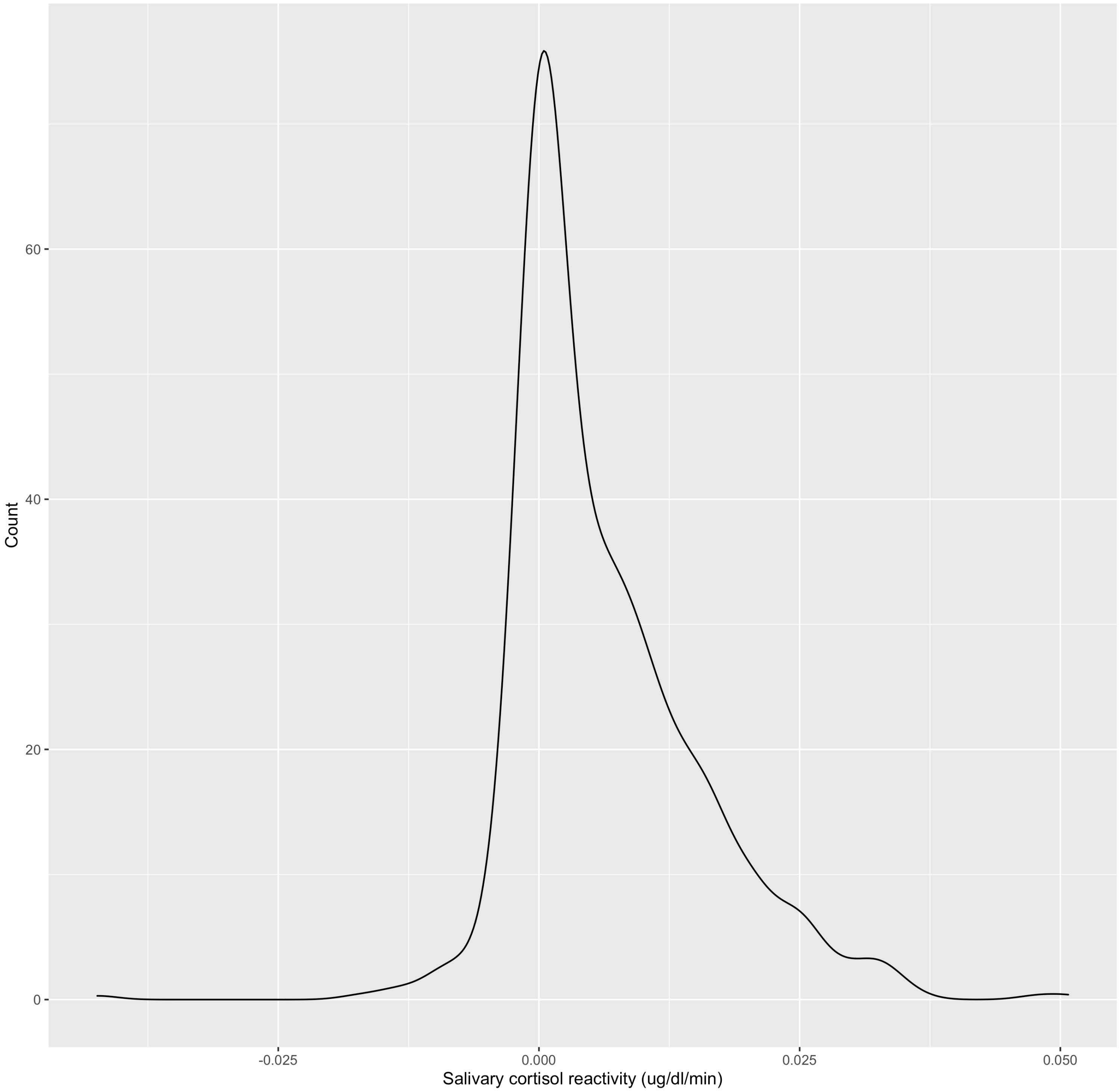

Supplemental Figure 3. Spline curves of the associations between HPA axis biomarkers and child development at Year 2.

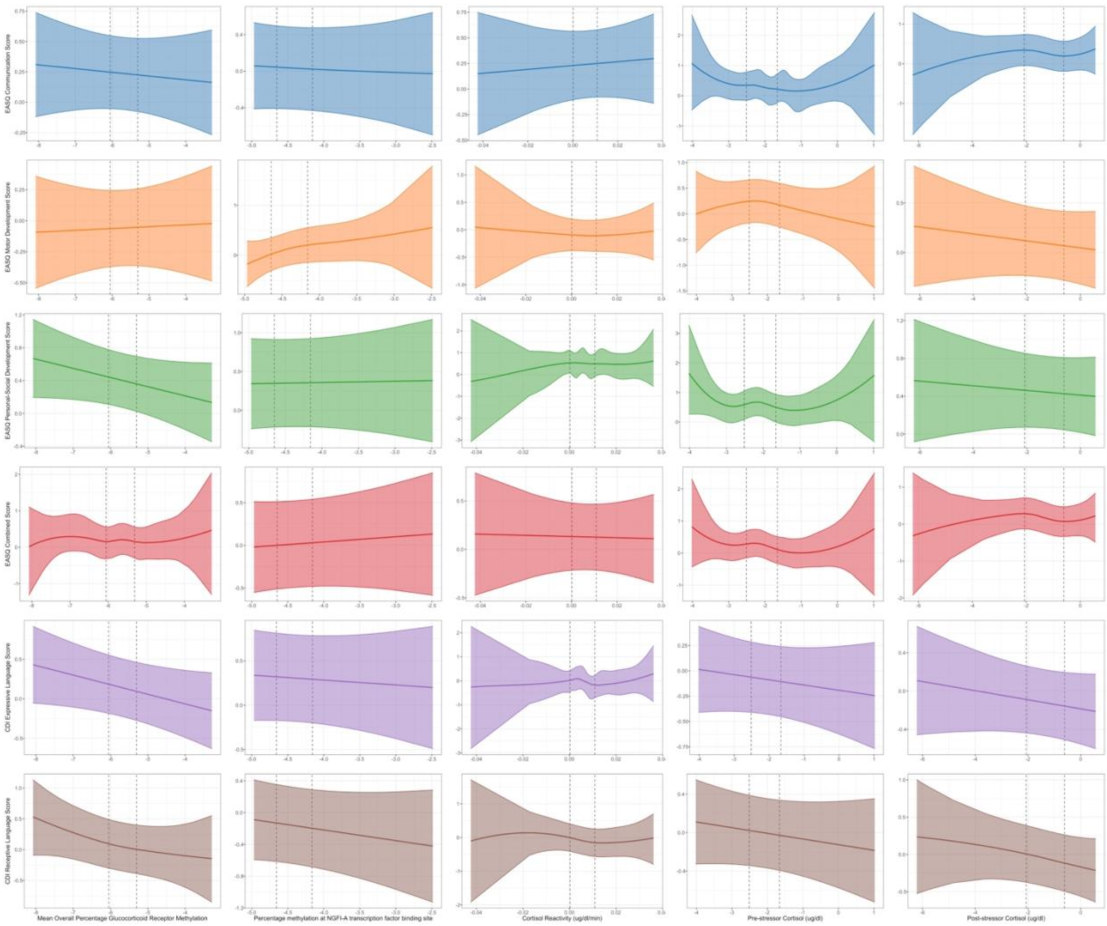

Supplemental Figure 4. Spline curves of the associations between concurrent urinary F2 isoprostanes and child development at Year 1.

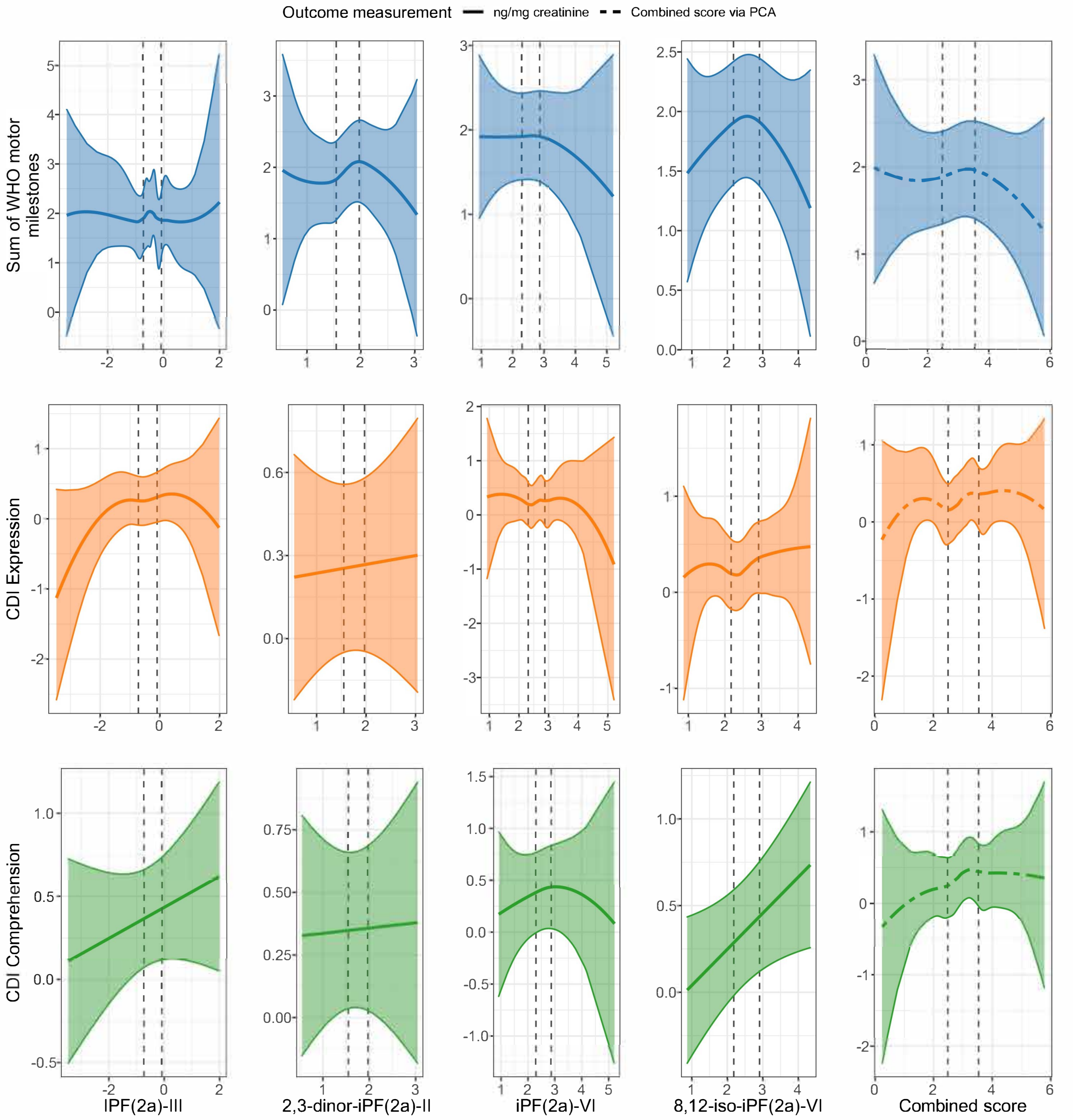

Supplemental Table 1. Study exposures, outcomes, and hypothesized relationships

Exposure Biological Domain and Biomarkers	Outcomes	Hypothesis
HPA axis: Cortisol via concentrations pre-stressor, post-stressor, and reactivity at Year 2	CDI and EASQ at Year 2	Cortisol concentrations and reactivity are positive correlated with child development
HPA axis: Glucocorticoid receptor methylation via percentage methylation at NGFI-A transcription factor binding site and mean overall glucocorticoid receptor methylation at Year 2	CDI and EASQ at Year 2	Glucocorticoid receptor methylation is negatively correlated with child development
SAM axis: Salivary alpha-amylase (concentrations pre-stressor, post-stressor, and reactivity), mean arterial pressure and resting heart rate at Year 2	CDI and EASQ at Year 2	SAM axis biomarkers are negatively associated with child development
Oxidative status: Individual urinary F2 isoprostane isomers and combined score at Year 1	WHO motor milestones and CDI at Year 1; CDI and EASQ at Year 2	Oxidative status is inversely associated with child development

CDI: MacArthur-Bates Communicative Development Inventories; EASQ: Extended Ages and Stages Questionnaire

Supplemental Table 2. Hypothalamic-pituitary-adrenal axis biomarkers and child development at Year 2

Exposure	Outcome	N	25th Percentile	75th Percentile	Outcome, 75th Percentile v. 25th Percentile			
					Adjusted			
					Predicted Outcome at 25th Percentile	Predicted Outcome at 75th Percentile	Coefficient (95% CI)	P-value
Cortisol reactivity (μg/dl/min)	EASQ communication score	545	0	0.01	0.23	0.25	0.02 (-0.07, 0.11)	0.67
	EASQ gross motor score	538	0	0.01	-0.09	-0.11	-0.01 (-0.13, 0.1)	0.81
	EASQ personal social score	544	0	0.01	0.57	0.43	-0.14 (-0.4, 0.12)	0.3
	Combined EASQ score	543	0	0.01	0.13	0.13	-0.01 (-0.1, 0.09)	0.9
	CDI expressive language score	552	0	0.01	-0.01	-0.24	-0.23 (-0.45, 0)	0.05
	CDI comprehension score	547	0	0.01	-0.01	-0.16	-0.15 (-0.29, -0.01)	0.04
Pre-stressor cortisol (μg/dl)	EASQ communication score	588	-2.51	-1.66	0.32	0.24	-0.07 (-0.32, 0.17)	0.57
	EASQ gross motor score	582	-2.51	-1.66	0.24	0.17	-0.07 (-0.21, 0.07)	0.31
	EASQ personal social score	588	-2.51	-1.66	0.57	0.47	-0.1 (-0.33, 0.12)	0.38
	Combined EASQ score	587	-2.51	-1.66	0.27	0.09	-0.17 (-0.38, 0.03)	0.1
	CDI expressive language score	595	-2.51	-1.66	-0.06	-0.11	-0.04 (-0.13, 0.05)	0.35
	CDI comprehension score	591	-2.51	-1.66	0.02	-0.03	-0.05 (-0.14, 0.04)	0.28
Post-stressor cortisol (μg/dl)	EASQ communication score	550	-2.08	-0.61	0.34	0.19	-0.16 (-0.32, 0.01)	0.06

Exposure	Outcome	N	25th Percentile	75th Percentile	Outcome, 75th Percentile v. 25th Percentile			
					Adjusted			
					Predicted Outcome at 25th Percentile	Predicted Outcome at 75th Percentile	Coefficient (95% CI)	P-value
	EASQ gross motor score	543	-2.06	-0.61	0.12	0.07	-0.05 (-0.17, 0.07)	0.42
	EASQ personal social score	549	-2.08	-0.61	0.46	0.43	-0.04 (-0.16, 0.09)	0.59
	Combined EASQ score	548	-2.07	-0.61	0.29	0.07	-0.22 (-0.41, -0.04)	0.02
	CDI expressive language score	557	-2.06	-0.61	-0.09	-0.16	-0.07 (-0.18, 0.04)	0.24
	CDI comprehension score	552	-2.07	-0.61	0.01	-0.12	-0.12 (-0.24, -0.01)	0.04
Mean overall percentage glucocorticoid receptor methylation	EASQ communication score	557	-6.05	-5.31	0.25	0.22	-0.02 (-0.1, 0.06)	0.58
	EASQ gross motor score	551	-6.05	-5.3	-0.06	-0.05	0.01 (-0.08, 0.1)	0.82
	EASQ personal social score	557	-6.05	-5.31	0.44	0.36	-0.08 (-0.17, 0.01)	0.07
	Combined EASQ score	556	-6.05	-5.3	0.12	0.13	0.01 (-0.21, 0.23)	0.95
	CDI expressive language score	563	-6.05	-5.3	0.18	0.09	-0.09 (-0.17, -0.01)	0.03
	CDI comprehension score	559	-6.05	-5.3	0.1	0.01	-0.09 (-0.19, 0.01)	0.07
Percentage methylation at NGFI-A transcription factor binding site (CpG site #12)	EASQ communication score	339	-4.65	-4.16	0.04	0.02	-0.02 (-0.13, 0.09)	0.71
	EASQ gross motor score	333	-4.65	-4.16	0.02	0.2	0.18 (0, 0.37)	0.05

Exposure	Outcome	N	25th Percentile	75th Percentile	Outcome, 75th Percentile v. 25th Percentile			
					Adjusted			
					Predicted Outcome at 25th Percentile	Predicted Outcome at 75th Percentile	Coefficient (95% CI)	P-value
	EASQ personal social score	339	-4.65	-4.16	0.35	0.36	0.01 (-0.11, 0.13)	0.9
	Combined EASQ score	338	-4.65	-4.16	0	0.03	0.03 (-0.08, 0.14)	0.62
	CDI expressive language score	342	-4.66	-4.16	0.32	0.29	-0.03 (-0.14, 0.08)	0.63
	CDI comprehension score	339	-4.66	-4.16	-0.13	-0.2	-0.07 (-0.18, 0.05)	0.26

Analyses adjusted for child age and child sex, and screened the following covariates for potential inclusion (see Appendix 3 for details) -- child birth order, maternal age, maternal height, maternal education, household food insecurity, number of children in the household, number of individuals living in the compound, distance to primary drinking water source, household assets, prior anthropometry, month of assessment, treatment arm, pre-stressor sample collection time, maternal Center for Epidemiologic Studies Depression Revised Scale score, maternal Perceived Stress Scale score, and maternal lifetime cumulative exposure to intimate partner violence.

* P-value < 0.2 after adjusting for multiple comparisons using the Benjamini-Hochberg procedure

Supplemental Table 3. Sympathetic adrenomedullary axis biomarkers and child development at Year 2

Exposure	Outcome	N	25th Percentile	75th Percentile	Outcome, 75th Percentile v. 25th Percentile			
					Adjusted			
					Predicted Outcome at 25th Percentile	Predicted Outcome at 75th Percentile	Coefficient (95% CI)	P-value
Salivary alpha-amylase reactivity (U/ml/min)	EASQ communication score	561	-0.08	4.61	0.11	0.13	0.02 (-0.04, 0.07)	0.57
	EASQ gross motor score	555	-0.08	4.54	-0.08	-0.05	0.04 (-0.03, 0.1)	0.26
	EASQ personal social score	560	-0.08	4.61	0.32	0.37	0.06 (-0.18, 0.3)	0.65
	Combined EASQ score	559	-0.08	4.6	0.03	0.18	0.15 (-0.06, 0.36)	0.16
	CDI expressive language score	568	-0.08	4.52	-0.2	-0.18	0.02 (-0.03, 0.08)	0.45
	CDI comprehension score	564	-0.08	4.49	-0.05	-0.02	0.03 (-0.03, 0.09)	0.29
Pre-stressor salivary alpha-amylase (U/ml)	EASQ communication score	584	3.5	4.64	0.25	0.17	-0.09 (-0.18, 0.01)	0.07
	EASQ gross motor score	578	3.5	4.65	0.04	-0.14	-0.18 (-0.35, 0)	0.04
	EASQ personal social score	584	3.5	4.65	0.45	0.43	-0.02 (-0.23, 0.19)	0.84
	Combined EASQ score	583	3.5	4.65	0.23	0.04	-0.19 (-0.4, 0.02)	0.07
	CDI expressive language score	591	3.51	4.64	-0.1	-0.16	-0.07 (-0.22, 0.08)	0.39
	CDI comprehension score	587	3.5	4.65	0.05	0.01	-0.04 (-0.15, 0.06)	0.42

Exposure	Outcome	N	25th Percentile	75th Percentile	Outcome, 75th Percentile v. 25th Percentile			
					Adjusted			
					Predicted Outcome at 25th Percentile	Predicted Outcome at 75th Percentile	Coefficient (95% CI)	P-value
Post-stressor salivary alpha-amylase (U/ml)	EASQ communication score	571	3.94	5.17	0.22	0.22	0 (-0.1, 0.09)	0.98
	EASQ gross motor score	565	3.94	5.16	0.21	0.19	-0.02 (-0.12, 0.09)	0.78
	EASQ personal social score	570	3.94	5.17	0.45	0.47	0.02 (-0.08, 0.13)	0.66
	Combined EASQ score	569	3.94	5.17	0.18	0.19	0.01 (-0.09, 0.11)	0.9
	CDI expressive language score	578	3.94	5.16	-0.19	-0.16	0.03 (-0.06, 0.13)	0.53
	CDI comprehension score	574	3.94	5.16	-0.08	-0.04	0.04 (-0.05, 0.14)	0.37
Mean arterial pressure (mmHg)	EASQ communication score	1196	60.89	69	-0.18	-0.13	0.04 (-0.04, 0.12)	0.29
	EASQ gross motor score	1176	60.89	69	0.38	0.4	0.02 (-0.04, 0.09)	0.49
	EASQ personal social score	1194	60.89	69	0.04	-0.04	-0.08 (-0.26, 0.09)	0.35
	Combined EASQ score	1191	60.89	69	0.07	0.09	0.02 (-0.14, 0.17)	0.85
	CDI expressive language score	1209	60.89	69	-0.02	0.07	0.09 (-0.08, 0.25)	0.31
	CDI comprehension score	1195	60.94	69	0.02	0.01	-0.01 (-0.16, 0.15)	0.92
Mean resting heart rate (bpm)	EASQ communication score	1198	99.33	118.33	-0.15	-0.15	-0.01 (-0.07, 0.06)	0.89

Exposure	Outcome	N	25th Percentile	75th Percentile	Outcome, 75th Percentile v. 25th Percentile			
					Adjusted			
					Predicted Outcome at 25th Percentile	Predicted Outcome at 75th Percentile	Coefficient (95% CI)	P-value
	EASQ gross motor score	1178	99.33	118.33	0.39	0.37	-0.02 (-0.08, 0.05)	0.58
	EASQ personal social score	1196	99.33	118.33	0.03	-0.07	-0.1 (-0.25, 0.06)	0.23
	Combined EASQ score	1193	99.33	118.33	-0.09	-0.11	-0.02 (-0.1, 0.06)	0.68
	CDI expressive language score	1211	99.33	118.5	0.01	-0.03	-0.04 (-0.11, 0.02)	0.18
	CDI comprehension score	1196	99.33	118.33	-0.01	-0.04	-0.03 (-0.09, 0.03)	0.27

Analyses adjusted for child age and child sex, and screened the following covariates for potential inclusion (see Appendix 3 for details) -- child birth order, maternal age, maternal height, maternal education, household food insecurity, number of children in the household, number of individuals living in the compound, distance to primary drinking water source, household assets, prior anthropometry, month of assessment, treatment arm, pre-stressor sample collection time, maternal Center for Epidemiologic Studies Depression Revised Scale score, maternal Perceived Stress Scale score, and maternal lifetime cumulative exposure to intimate partner violence.

* P-value < 0.2 after adjusting for multiple comparisons using the Benjamini-Hochberg procedure

Supplemental Table 4. F2-isoprostanes and child development at Year 1

Exposure	Outcome	N	25th Percentile	75th Percentile	Outcome, 75th Percentile v. 25th Percentile			
					Adjusted			
					Predicted Outcome at 25th Percentile	Predicted Outcome at 75th Percentile	Coefficient (95% CI)	P-value
iPF(2α)-III (ng/mg creatinine)	Sum of 2nd, 4th, 5th, and 6th WHO motor milestones	571	-0.73	-0.09	1.84	1.79	-0.05 (-0.37, 0.27)	0.77
	CDI expressive language Z-score	672	-0.72	-0.09	0.26	0.31	0.05 (-0.1, 0.2)	0.52
	CDI comprehension Z-score	576	-0.73	-0.09	0.37	0.43	0.06 (-0.04, 0.16)	0.24
2,3-dinor-iPF(2α)-III (ng/mg creatinine)	Sum of 2nd, 4th, 5th, and 6th WHO motor milestones	571	1.54	1.97	1.81	2.09	0.27 (0.04, 0.51)	0.02
	CDI expressive language Z-score	672	1.55	1.97	0.25	0.27	0.01 (-0.09, 0.11)	0.8
	CDI comprehension Z-score	576	1.55	1.97	0.35	0.36	0.01 (-0.11, 0.12)	0.89
iPF(2α)-VI (ng/mg creatinine)	Sum of 2nd, 4th, 5th, and 6th WHO motor milestones	571	2.3	2.87	1.92	1.92	0 (-0.15, 0.15)	0.98
	CDI expressive language Z-score	672	2.31	2.87	0.2	0.22	0.02 (-0.2, 0.24)	0.86
	CDI comprehension Z-score	576	2.3	2.87	0.38	0.43	0.05 (-0.08, 0.18)	0.43

Exposure	Outcome	N	25th Percentile	75th Percentile	Outcome, 75th Percentile v. 25th Percentile			
					Adjusted			
					Predicted Outcome at 25th Percentile	Predicted Outcome at 75th Percentile	Coefficient (95% CI)	P-value
8,12-iso-iPF(2α)-VI (ng/mg creatinine)	Sum of 2nd, 4th, 5th, and 6th WHO motor milestones	571	2.17	2.91	1.9	1.91	0.01 (-0.17, 0.19)	0.94
	CDI expressive language Z-score	672	2.17	2.92	0.19	0.36	0.17 (-0.03, 0.38)	0.09
	CDI comprehension Z-score	576	2.18	2.91	0.29	0.44	0.15 (0.04, 0.27)	0.01
Combined F2-isoprostanes score	Sum of 2nd, 4th, 5th, and 6th WHO motor milestones	571	2.48	3.54	1.88	1.96	0.09 (-0.11, 0.29)	0.41
	CDI expressive language Z-score	672	2.5	3.55	0.1	0.31	0.21 (-0.03, 0.44)	0.08
	CDI comprehension Z-score	576	2.48	3.54	0.22	0.4	0.18 (-0.08, 0.44)	0.18

Analyses adjusted for child age and child sex, and screened the following covariates for potential inclusion (see Appendix 3 for details) -- child birth order, maternal age, maternal height, maternal education, household food insecurity, number of children in the household, number of individuals living in the compound, distance to primary drinking water source, household assets, prior anthropometry, month of assessment, treatment arm, pre-stressor sample collection time, maternal Center for Epidemiologic Studies Depression Revised Scale score, maternal Perceived Stress Scale score, and maternal lifetime cumulative exposure to intimate partner violence.

* P-value < 0.2 after adjusting for multiple comparisons using the Benjamini-Hochberg procedure

Supplemental Table 5. F2-isoprostanes at Year 1 and child development at Year 2

Exposure	Outcome	N	25th Percentile	75th Percentile	Outcome, 75th Percentile v. 25th Percentile			
					Adjusted			
					Predicted Outcome at 25th Percentile	Predicted Outcome at 75th Percentile	Coefficient (95% CI)	P-value
iPF(2α)-III (ng/mg creatinine)	EASQ communication score	551	-0.74	-0.09	0.19	0.21	0.01 (-0.07, 0.1)	0.79
	EASQ gross motor score	546	-0.73	-0.09	0.32	0.24	-0.08 (-0.18, 0.01)	0.08
	EASQ personal social score	551	-0.73	-0.09	0.42	0.41	0 (-0.12, 0.11)	0.97
	Combined EASQ score	550	-0.73	-0.09	0.23	0.21	-0.01 (-0.1, 0.08)	0.8
	CDI expressive language score	558	-0.74	-0.09	0	0.05	0.04 (-0.04, 0.13)	0.34
	CDI comprehension score	554	-0.73	-0.09	0.07	0.13	0.06 (-0.03, 0.15)	0.21
2,3-dinor-iPF(2α)-III (ng/mg creatinine)	EASQ communication score	551	1.54	1.97	0.21	0.17	-0.04 (-0.15, 0.07)	0.49
	EASQ gross motor score	546	1.54	1.97	0.37	0.29	-0.08 (-0.34, 0.18)	0.55
	EASQ personal social score	551	1.54	1.97	0.42	0.35	-0.07 (-0.22, 0.08)	0.38
	Combined EASQ score	550	1.54	1.97	0.21	0.12	-0.09 (-0.22, 0.05)	0.21
	CDI expressive language score	558	1.54	1.97	0.01	-0.03	-0.04 (-0.18, 0.1)	0.6
	CDI comprehension score	554	1.54	1.97	0.08	0.05	-0.03 (-0.15, 0.09)	0.64
iPF(2α)-VI (ng/mg creatinine)	EASQ communication score	551	2.3	2.87	0.18	0.1	-0.08 (-0.21, 0.04)	0.19

Exposure	Outcome	N	25th Percentile	75th Percentile	Outcome, 75th Percentile v. 25th Percentile			
					Adjusted			
					Predicted Outcome at 25th Percentile	Predicted Outcome at 75th Percentile	Coefficient (95% CI)	P-value
	EASQ gross motor score	546	2.31	2.87	0.33	0.27	-0.07 (-0.17, 0.04)	0.22
	EASQ personal social score	551	2.31	2.87	0.42	0.28	-0.14 (-0.25, -0.03)	0.01
	Combined EASQ score	550	2.3	2.87	0.22	0.12	-0.1 (-0.23, 0.04)	0.15
	CDI expressive language score	558	2.3	2.87	0.01	-0.07	-0.08 (-0.17, 0.02)	0.12
	CDI comprehension score	554	2.31	2.87	0.06	0.05	-0.01 (-0.15, 0.13)	0.91
8,12-iso-iPF(2α)-VI (ng/mg creatinine)	EASQ communication score	551	2.16	2.91	0.2	0.26	0.06 (-0.07, 0.19)	0.36
	EASQ gross motor score	546	2.17	2.91	0.29	0.31	0.02 (-0.09, 0.13)	0.76
	EASQ personal social score	551	2.17	2.91	0.41	0.42	0 (-0.12, 0.12)	0.97
	Combined EASQ score	550	2.17	2.91	0.21	0.25	0.04 (-0.1, 0.17)	0.6
	CDI expressive language score	558	2.17	2.91	0.01	-0.02	-0.03 (-0.22, 0.16)	0.76
	CDI comprehension score	554	2.17	2.91	0.03	0.09	0.05 (-0.13, 0.24)	0.57
Combined F2-isoprostanes score	EASQ communication score	551	2.48	3.56	0.19	0.19	-0.01 (-0.11, 0.1)	0.9
	EASQ gross motor score	546	2.49	3.55	0.32	0.27	-0.06 (-0.17, 0.05)	0.31
	EASQ personal social score	551	2.48	3.56	0.42	0.38	-0.04 (-0.16, 0.08)	0.49

Exposure	Outcome	N	25th Percentile	75th Percentile	Outcome, 75th Percentile v. 25th Percentile			
					Adjusted			
					Predicted Outcome at 25th Percentile	Predicted Outcome at 75th Percentile	Coefficient (95% CI)	P-value
	Combined EASQ score	550	2.48	3.55	0.23	0.19	-0.04 (-0.15, 0.08)	0.54
	CDI expressive language score	558	2.47	3.55	0	0.02	0.02 (-0.08, 0.12)	0.72
	CDI comprehension score	554	2.49	3.54	0.05	0.11	0.06 (-0.05, 0.17)	0.28

Analyses adjusted for child age and child sex, and screened the following covariates for potential inclusion (see Appendix 3 for details) -- child birth order, maternal age, maternal height, maternal education, household food insecurity, number of children in the household, number of individuals living in the compound, distance to primary drinking water source, household assets, prior anthropometry, month of assessment, treatment arm, pre-stressor sample collection time, maternal Center for Epidemiologic Studies Depression Revised Scale score, maternal Perceived Stress Scale score, and maternal lifetime cumulative exposure to intimate partner violence.

* P-value < 0.2 after adjusting for multiple comparisons using the Benjamini-Hochberg procedure

Supplemental Table 6. F2-isoprostanes and time to WHO motor milestone at Year 1

Exposure	Outcome	N	25th Percentile	75th Percentile	Outcome, 75th Percentile v. 25th Percentile	
					Adjusted	
					Hazard Ratio (95% CI)	P-value
iPF(2α)-III (ng/mg creatinine)	Time to sitting unsupported	577	-0.73	-0.09	0 (0, 7.2e+17)	0.61
	Time to crawling	682	-0.72	-0.09	1.02 (0.91, 1.14)	0.76
	Time to standing with support	671	-0.72	-0.1	1.01 (0.88, 1.17)	0.86
	Time to walking with support	670	-0.72	-0.1	1.03 (0.9, 1.17)	0.72
	Time to standing unsupported	672	-0.72	-0.09	0.95 (0.65, 1.39)	0.79
	Time to walking unsupported	679	-0.72	-0.09	1.05 (0.88, 1.25)	0.6
2,3-dinor- iPF(2α)-III (ng/mg creatinine)	Time to sitting unsupported	577	1.55	1.97	0 (0, 1.18e+59)	0.76
	Time to crawling	682	1.55	1.97	1 (0.72, 1.37)	0.98
	Time to standing with support	671	1.55	1.97	0.95 (0.81, 1.11)	0.55
	Time to walking with support	670	1.55	1.97	0.97 (0.82, 1.15)	0.72
	Time to standing unsupported	672	1.55	1.97	1.12 (0.93, 1.33)	0.23
	Time to walking unsupported	679	1.55	1.97	1 (0.81, 1.23)	1
iPF(2α)-VI (ng/mg creatinine)	Time to sitting unsupported	577	2.3	2.87	6.2e+8 (0, 8.7e+65)	0.78
	Time to crawling	682	2.31	2.87	1 (0.89, 1.14)	0.97
	Time to standing with support	671	2.31	2.87	0.91 (0.78, 1.06)	0.22
	Time to walking with support	670	2.31	2.87	1.12 (0.88, 1.42)	0.35
	Time to standing unsupported	672	2.31	2.87	0.8 (0.51, 1.26)	0.35

Exposure	Outcome	N	25th Percentile	75th Percentile	Outcome, 75th Percentile v. 25th Percentile	
					Adjusted	
					Hazard Ratio (95% CI)	P-value
	Time to walking unsupported	679	2.31	2.87	1.02 (0.76, 1.37)	0.88
8,12-iso-iPF(2α)-VI (ng/mg creatinine)	Time to sitting unsupported	577	2.19	2.91	0.03 (0, 2.6e+15)	0.87
	Time to crawling	682	2.17	2.92	0.98 (0.86, 1.12)	0.79
	Time to standing with support	671	2.16	2.91	0.88 (0.74, 1.04)	0.12
	Time to walking with support	670	2.16	2.92	0.99 (0.8, 1.23)	0.95
	Time to standing unsupported	672	2.16	2.92	1.07 (0.79, 1.45)	0.69
	Time to walking unsupported	679	2.17	2.91	1.12 (0.81, 1.53)	0.51
Combined F2-isoprostanes score	Time to sitting unsupported	577	2.5	3.55	0 (0, 5.2e+14)	0.52
	Time to crawling	682	2.49	3.54	0.99 (0.87, 1.14)	0.93
	Time to standing with support	671	2.48	3.54	1.12 (0.75, 1.69)	0.59
	Time to walking with support	670	2.48	3.54	1 (0.84, 1.2)	0.98
	Time to standing unsupported	672	2.48	3.54	1.2 (0.8, 1.82)	0.38
	Time to walking unsupported	679	2.49	3.54	0.98 (0.79, 1.22)	0.87

Analyses adjusted for child age and child sex, and screened the following covariates for potential inclusion (see Appendix 3 for details) -- child birth order, maternal age, maternal height, maternal education, household food insecurity, number of children in the household, number of individuals living in the compound, distance to primary drinking water source, household assets, prior anthropometry, month of assessment, treatment arm, pre-stressor sample collection time, maternal Center for Epidemiologic Studies Depression Revised Scale score, maternal Perceived Stress Scale score, and maternal lifetime cumulative exposure to intimate partner violence.

*P-value < 0.2 after adjusting for multiple comparisons using the Benjamini-Hochberg procedure

Hazard ratio could not be estimated for sitting without support since nearly all children had achieved this milestone before time of measurement

REFERENCES

1. Lin, A. *et al.* Effects of Water, Sanitation, Handwashing, and Nutritional Interventions on Environmental Enteric Dysfunction in Young Children: A Cluster-randomized, Controlled Trial in Rural Bangladesh. *Clinical Infectious Diseases* **70**, 738–747 (2020).
2. Il'yasova, D. *et al.* Urinary biomarkers of oxidative status in a clinical model of oxidative assault. *Cancer Epidemiol Biomarkers Prev* **19**, 1506–1510 (2010).
3. Nater, U. M. & Rohleder, N. Salivary alpha-amylase as a non-invasive biomarker for the sympathetic nervous system: current state of research. *Psychoneuroendocrinology* **34**, 486–496 (2009).
4. Jansen, J., Beijers, R., Riksen-Walraven, M. & de Weerth, C. Cortisol reactivity in young infants. *Psychoneuroendocrinology* **35**, 329–338 (2010).
5. Tost, J. & Gut, I. G. DNA methylation analysis by pyrosequencing. *Nat Protoc* **2**, 2265–2275 (2007).
6. Tofail, F. *et al.* Effect of water quality, sanitation, hand washing, and nutritional interventions on child development in rural Bangladesh (WASH Benefits Bangladesh): a cluster-randomised controlled trial. *The Lancet Child & Adolescent Health* **2**, 255–268 (2018).
7. Hamadani, J. D. *et al.* Validity and Reliability of Mothers' Reports of Language Development in 1-Year-Old Children in a Large-Scale Survey in Bangladesh. *Food Nutr Bull* **31**, S198–S206 (2010).
8. WHO Motor Development Study: windows of achievement for six gross motor development milestones. *Acta Paediatr Suppl* **450**, 86–95 (2006).
9. Fernald, L. C. H., Kariger, P., Hidrobo, M. & Gertler, P. J. Socioeconomic gradients in child development in very young children: evidence from India, Indonesia, Peru, and Senegal. *Proc Natl Acad Sci U S A* **109 Suppl 2**, 17273–17280 (2012).

10. Small, J. W., Hix-Small, H., Vargas-Baron, E. & Marks, K. P. Comparative use of the Ages and Stages Questionnaires in low- and middle-income countries. *Dev Med Child Neurol* **61**, 431–443 (2019).

11. Radloff, L. S. The CES-D Scale: A Self-Report Depression Scale for Research in the General Population. *Applied Psychological Measurement* **1**, 385–401 (1977).

12. Cohen, S., Kamarck, T. & Mermelstein, R. A global measure of perceived stress. *J Health Soc Behav* **24**, 385–396 (1983).

13. Garcia-Moreno, C. *et al. WHO multi-country study study on women's health and domestic violence against women: initial results on prevalence, health outcomes and women's responses*. (World Health Organization, 2005).

14. Lin, A., Mertens, A., Li, A., Butzin-Dozier, Z. & Tan, S. WASH Benefits Bangladesh Child Stress and Child Development Analysis Plan. (2021).
